# Free Radicals in Health and Disease

**DOI:** 10.1002/mco2.70396

**Published:** 2025-09-21

**Authors:** Xiaofeng Dai, Zizheng Huang, Ruohan Lyu

**Affiliations:** ^1^ The First Affiliated Hospital of Xi'an Jiaotong University Xi'an Jiaotong University Xi'an China; ^2^ Department of Statistics Beijing Forestry University Beijing China

**Keywords:** cancer, cold atmospheric plasma, degeneration disease, free radicals, oxidative stress, reductive stress

## Abstract

Free radicals, molecules with unpaired electrons, are double‐edged swords. While they may cause damages to cells and threaten human health, they play essential roles in cellular signaling toward mitochondrial and immune homeostasis. Overproduction or insufficient supply of free radicals can both lead to health concerns and disease syndromes by causing oxidative or reductive stress to cells. Current redox therapies frequently fail clinically due to imprecise dosing and targeting, causing therapeutic futility or paradoxical harm by disrupting redox homeostasis, necessitating integrated frameworks linking redox biology to precision interventions alongside therapeutic innovation. This review explores free radicals’ generation sources, characterizes mitochondrial oxidative phosphorylation and pathological hyperglycemia as pivotal endogenous sources, and proposes oxygen and transition metals as fundamental regulators. This paper synthesizes multidimensional molecular mechanisms and pathologies arising from redox dysregulation and establishes reductive stress as a critical pathogenesis driver alongside oxidative stress. This review discusses free radical approaches and proposes cold atmospheric plasma as a transformative redox‐modulating technology capable of bridging therapeutic dichotomies through calibrated interventions. By integrating mechanistic insights with innovative methodologies, this work underscores the imperative to innovatively harness the dual nature of free radicals for precision health and disease management.

## Graphical abstract

Free radicals have dual roles that can either selectively create oxidative damages to transformed cells in treating proliferative diseases such as cancers and some dermatological disorders or boost the antioxidant system in treating degenerative diseases such as neurodegenerative disorder, joint degeneration, and metabolic conditions. CAP can be used to treat both types of diseases as its free radical concentration fall into the low to moderate range that can be tuned for restored cellular redox homeostasis.

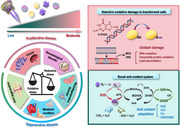

## 1 Introduction

Free radicals, defined as molecules or atoms possessing one or more unpaired electrons in the outermost electron shell, exhibit high reactivity and ephemeral lifespans, with reactive oxygen species (ROS) and reactive nitrogen species (RNS) being the most biologically significant subsets. Free radicals can either cause cell damages or participate in cell signaling, the effect of which depends on their spatiotemporal generation and concentration. At physiological levels, free radicals are involved in signaling pathways that regulate mitochondrial dynamics [1], biogenesis [2], turnover [3] for promoted metabolic resilience; and participate in the innate immune response to eliminate pathogens, and regulate the differentiation, activation, and proliferation of primary players in the adaptive immune system [4, 5]. However, when overproduced, these radicals can overwhelm the body's antioxidant defenses, leading to the oxidative stress and subsequent damages to lipids, proteins, and nucleic acids [5]. This imbalance, either in excessive production or insufficient supply, is implicated in numerous pathological conditions, including proliferative syndromes such as cancers [6] and psoriasis [7], degenerative disorders such as neurodegeneration [8] and joint degenerative diseases [9], as well as metabolic diseases like diabetes [10]. Since both under‐ and overproduction of free radicals can cause pathological conditions by introducing reductive or oxidative stress, therapeutic strategies targeting free radicals have shown great promises in mitigating these conditions. Accordingly, various antioxidant nutrient supplements, lifestyle recommendations, and pharmacological interventions aiming at modulating redox homeostasis for health and disease management have evolved. Yet, current redox‐editing modalities especially antioxidants often fail clinically [11, 12, 13, 14, 15, 16, 17, 18] due to imprecise targeting of pathological redox states and suboptimal dosing that inadequately modulate intracellular redox homeostasis, resulting in either therapeutic futility or paradoxical harm. Such ambiguities prevent restoration of redox equilibrium, allowing persistent oxidative damage or reductive stress that exacerbates cellular injury. Thus, integrated frameworks linking molecular mechanisms to precision interventions are urgently required for context‐specific radical dosing, dynamic modulation of redox fluxes across disease stages, and therapeutic innovation.

Recent technological advancements have introduced novel approaches to harness the therapeutic potential of free radicals. Cold atmospheric plasma (CAP) is an emerging technology that generates a controlled stream of free radicals such as hydroxyl radicals (OH·), superoxide anion (O_2_^•−^), singlet delta oxygen (^1^O_2_), hydrogen peroxide (H_2_O_2_), ozone (O_3_), atomic nitrogen (N), various nitrogen oxides (e.g., NO, NO_2_), and nitric acid (HNO_3_) at the room temperatures [19]. CAP has demonstrated a great potential in various medical applications such as wound healing [20], microbial ablation [21], and more recently, in treating complex diseases including cancers [22], psoriasis [23], and neurodegenerative syndromes [24]. By delivering a controlled dose of reactive species, CAP can induce selective death of diseased cells or enhance the tissue repair without causing significant damages to the surrounding healthy tissues, representing a versatile and innovative approach to leveraging the therapeutic benefits of free radicals.

This review bridges critical knowledge gaps by synthesizing the complexity of free radical biology with next‐generation therapeutic strategies. Contrasting static conventional approaches, this review highlights the transformative potential of CAP in dynamically restoring redox balance across pathological contexts by calibrating radical flux to disease‐specific pathophysiology that exemplifies a new era of precision medicine. Specifically, this review introduces the generation sources of free radicals in Section 2; emphasizes its representative pathways, duality nature, switching nexus, and biological impacts in Section 3; discusses pathological conditions caused by redox dis‐regulation in Section 4; introduces existing and emerging redox‐regulatory strategies for disease management in Section 5; and underpins emerging frontiers for improved precision redox intervention in Section 6 before concluding this paper.

## 2 Generation of Free Radicals

Free radicals originate from both endogenous metabolic processes and exogenous environmental sources. Their dysregulated production disrupts cellular homeostasis, a multidimensional equilibrium governing metabolic signaling and immune function that critically demarcates health from disease. This section delineates free radical generation sources along with the multilayer infrastructure controlling its homeostasis.

### 2.1 Endogenous Sources

**FIGURE 1:**
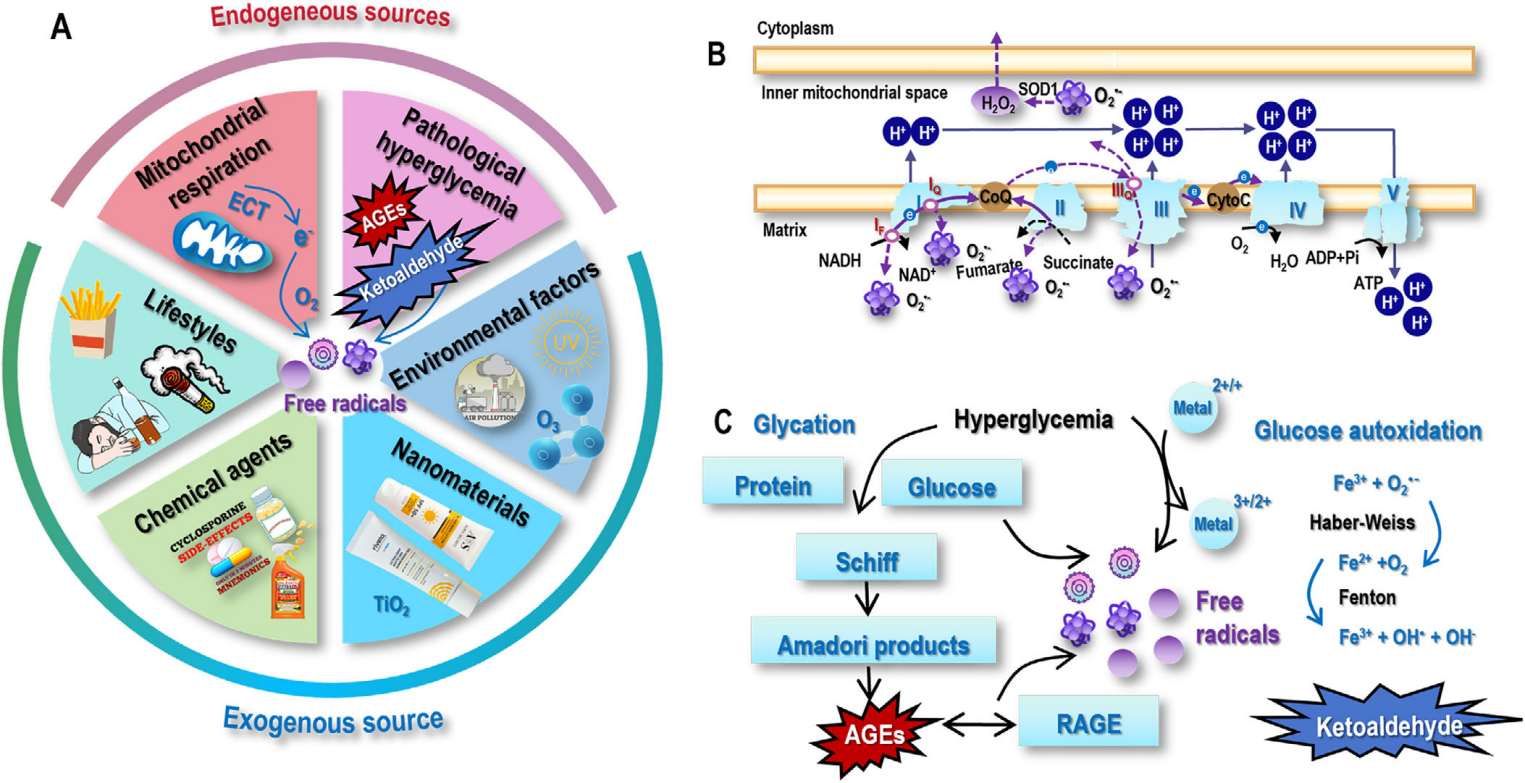
Primary endogenous and exogenous generation sources of free radicals. (A) Free radicals can come from both endogenous and exogenous generation sources. The primary sources for endogenous free radical generation include mitochondrial respiration and pathological hyperglycemia that underscore the vital roles of O_2_ and transition metals in driving endogenous free radical production. The major sources for exogenous free radical generation include lifestyles such as cigarette smoking, alcohol addiction, and unhealthy dietary, chemical agents such as chemotherapy and herbicides, nanomaterials such as titanium dioxide (TiO_2_) in the sunscreen, and environmental factors such as air pollution, UV radiation, and ozone (O_3_). (B) In mitochondria respiration, mitochondrial electron transport chain (ETC) is the primary endogenous source of free radicals that includes complexes I–IV, as well as the electron transporters ubiquinone (CoQ) and cytochrome *c* (cytoC) during mitochondrial respiration. There are two electron transport paths in the ETC, that is, complex I/III/IV and complex II/III/IV. While complex I/III/IV uses NADH as the substrate, complex II/III/IV adopts succinate as the substrate. ATP is produced by complex V using energy accumulated in the proton gradient that is generated across the inner membrane during the electron flow. During mitochondria respiration, complexes I and III are the main sites for ROS production, where ROS can be generated at both I_F_ (FMN site) and I_Q_ (CoQ binding site) sites of complex I and at III_Qo_ site (QH_2_ oxidation site) of complex III. (C) Hyperglycemia may generate free radicals via inducing glycation and glucose autoxidation. In glycation, glucose is added to the free amino groups of proteins through covalent binding, forming Schiff bases, Amadori products, and advanced glycation end products (AGEs). AGEs interact with the receptor of AGE (RAGE) to further enhance free radical generation. Glucose autoxidation produces free radicals under hyperglycemia through Fenton and Haber–Weiss reactions that are enabled by transition metals, with ketoaldehyde being the byproduct.

Free radicals are generated endogenously as natural byproducts of cellular metabolism, with mitochondrial respiration as the primary production source and pathological hyperglycemia being the major accelerating condition (Figure 1A).

#### 2.1.1 Mitochondrial Respiration

Mitochondrial electron transport chain (ETC) is the primary endogenous source of free radicals that includes complexes I–IV, as well as the electron transporters ubiquinone and cytochrome *c* during mitochondrial respiration. There are two electron transport paths in the ETC, which are complex I/III/IV and complex II/III/IV. While complex I/III/IV uses nicotinamide adenine dinucleotide (NADH) as the substrate, complex II/III/IV adopts succinic acid as the substrate. ATP is produced by complex V (also named ATP synthase) using energy accumulated in the proton gradient, which is generated across the inner membrane during the electron flow [25].

Under physiological conditions, 0.2–2% of the electrons directly leak out of the ETC during cellular respiration and interact with oxygen (O_2_) to produce O_2_^•−^ or H_2_O_2_ [26, 27, 28]. So far, 11 sites have been identified to be associated with electron leaky and endogenous ROS production in mammalian mitochondria, with complex I and complex III being the main sites [29, 30, 31].

Complex I, also called NADH–ubiquinone (CoQ) oxidoreductase, is the largest enzyme complex in the ETC and produces the majority of endogenous ROS. Complex I contains two domains, that is, the membrane arm embedded in the inner membranes and the matrix arm protruding into the matrix, to collectively transfer electrons from NADH to CoQ. While the membrane arm contains seven hydrophobic subunits, the matrix arm harbors seven core subunits and three sets of cofactors, that is, a flavin mononucleotide (FMN), seven to nine iron–sulfur (FeS) clusters, and the electron accepting FeS cluster. Electrons enter the ETC and are passed to CoQ via a chain of FeS clusters arranged from low to high according to the potential. ROS can be generated in the matrix at both I_F_ (FMN site) and I_Q_ (CoQ binding site) sites during the electron transfer in complex I, where CoQ is reduced to ubiquinol (QH2) [25] (Figure 1B).

Complex III is also referred to as CoQ–cytochrome *c* reductase that transfers the electrons carried by QH2 to cytochrome *c* and CoQ. Complex III is a symmetrical dimer containing 11 subunits per monomer, with catalytically active subunits being cytochrome *b*, cytochrome *c*, and a high‐potential FeS cluster. The electron transfer in complex III is accomplished by the Q‐cycle, where two CoQ binding sites on cytochrome *b* are involved. These are the QH_2_ oxidation site (Q_o_) that is related to the low potential cytochrome *b* (*b*_L_), and the QH_2_ reduction site (Q_i_) that is related to the high potential cytochrome *b* (*b*_H_). In the Q‐cycle, while an electron is transferred to the FeS cluster and to cytochrome *c*, a QH_2_ is oxidized to ubisemiquinone (QH^−^) at the Q_o_ site; the second electron is transferred by QH^−^ to cytochrome *b*_L_ and to cytochrome *b*_H_ and CoQ at the Q_i_ site, whereas the second QH_2_ molecule is oxidized at the Q_o_ site. During the Q‐cycle where one electron is transferred to cytochrome *c* and the other electron to CoQ, a single electron can be directly leaked from QH^−^ of the Q_o_ site to react with O_2_ and form ROS [25] (Figure 1B).

Of a particular importance is O_2_ that receives the leaky electron to produce highly reactive species, emphasizing the centrality of O_2_ in free radical generation and energy production homeostasis.

#### 2.1.2 Pathological Hyperglycemia

Hyperglycemia may lead to free radical generation through several mechanisms of action, with glycation and glucose autoxidation being the primary sources [32]. Glycation is a nonenzymatic reaction that occurs on proteins, lipids, and nucleic acids under high concentrations of monosaccharaides. By adding monosaccharaides to the free amino groups of these molecules through covalent binding, this chemical reaction forms different oxidant by‐products such as advanced glycation end products (AGEs), Schiff bases and Amadori products [33, 34]. Of the particular importance is AGEs and their interactions with the receptor of AGE (RAGE), where AGEs–RAGE cross links are known to enhance free radical generation via activating a number of molecular pathways such as nuclear factor kappa‐light‐chain‐enhancer of activated B cells (NFκB) [35]. Glucose autoxidation is a chemical transition metal‐catalyzed process that produces deleterious free radicals and ketoaldehyde compounds under hyperglycemia. It generates H_2_O_2_ and several biomarkers of the oxidative stress such as acrolein [32, 36].

The catalytic process of glycation and glucose autoxidation may both involve transition metals such as iron (Fe^2+^), copper (Cu^+^), and zinc (Zn^2+^), which produce free radicals through the Fenton and Haber–Weiss reactions [37]. In the Fenton reaction, Fe^2+^ and H2O2 react to produce Fe^3+^, OH·, and hydroxyl anion (OH^−^); and OH· further reacts with H_2_O_2_ to produce O_2_^•−^, H^+^, and H_2_O. In Haber–Weiss reaction, O_2_^•−^ reacts with H2O2 to produce OH·, OH^−^, and O_2_, where Fe^3+^ is reduced to Fe^2+^ [37] (Figure 1C). In neurodegenerative diseases such as Alzheimer's and Parkinson's diseases, increased levels of labile Fe^2+^, Cu^+^, and Zn^2+^ ions have been implicated in the exacerbation of oxidative damage [38, 39, 40].

Central to free radicals generated in response hyperglycemia is transition metal that transduces electrons in a reversible manner during the generation process of reactive species, emphasizing the centrality of metal ion homeostasis in maintaining cellular redox balance and its therapeutic potential.

### 2.2 Exogenous Sources

Primary sources for exogenous free radical generation include lifestyles, chemical agents, nanomaterials, and environmental factors (Figure 1A).

#### 2.2.1 Lifestyles

Lifestyles such as cigarette smoking, alcohol abuse, and dietary factors such as processed food intake contribute to exogenous sources of free radicals.

Cigarette contains >4000 chemicals, including quinones, aldehydes, and nitric oxide (NO•), which synergize to produce ONOO^−^ and lipid peroxides (Figure 1A). E‐cigarettes may also lead to ROS generation by producing formaldehyde and acrolein. It has been documented that acrolein in cigarette smoke induced atherosclerosis by triggering the oxidative stress [41]; and acrolein in E‐cigarette promoted DNA fragmentation in lung fibroblasts by inducing mitochondrial ROS (mtROS) [42].

Ethanol metabolism is a complex process that involves multiple enzymatic pathways, each contributing to the generation of free radicals and the depletion of cellular antioxidants (Figure 1A). One of the primary enzymes involved in ethanol metabolism is cytochrome P450 2E1 (CYP2E1) that catalyzes the oxidation of ethanol to acetaldehyde, resulting in the generation of O_2_^•−^ and H_2_O_2_. Further metabolism of acetaldehyde by aldehyde dehydrogenase produces NADH, which leads to the depletion of glutathione (GSH), a crucial cellular antioxidant, to exacerbate the oxidative stress. Chronic alcohol intake has been shown to impose profound negative impact on liver physiology, particularly in hepatocytes. It has been demonstrated that prolonged ethanol consumption in mice significantly increased mtROS production in hepatocytes, which was associated with activated NOD‐like receptor thermal protein domain associated protein 3 (NLRP3) inflammasome and impaired liver functionality [43, 44, 45]. Such an intricate interplay between ethanol metabolism and oxidative stress underscores the importance of targeting ROS production as potential therapeutic strategies for alcohol‐related liver diseases.

AGEs are a group of heterogeneous compounds formed through nonenzymatic glycation reactions between reducing sugars and free amino groups of proteins, lipids, or nucleic acids. These compounds are commonly generated during high‐temperature cooking methods such as grilling and frying that promote the Maillard reaction. The consumption of foods enriched with AGEs has been associated with increased oxidative stress and inflammation. It has been demonstrated that AGEs induced free radical production through activating RAGE, which is a pattern recognition receptor that, upon binding to AGEs, activates NADH phosphate hydrogen (NADPH) oxidase (NOX) and leads to increased oxidative stress [46, 47, 48, 49] (Figure 1A). In addition to AGEs, other compounds formed during high‐temperature cooking such as acrylamide also impose health risks. It has been revealed that acrylamide exposure generated free radicals that triggered systemic inflammation by damaging intestinal epithelial tight junctions in rats [50]. These findings have highlighted the potential health hazards associated with dietary choices [51].

#### 2.2.2 Chemical Agents

Chemotherapeutic agents are known to induce oxidative stress as part of their mechanisms of action, leading to significant cellular damage and cytotoxicity (Figure 1A). Doxorubicin (DOX) and cisplatin, two widely used chemotherapeutic drugs, are prime examples of how free radical generation contributes to the therapeutic efficacy and what side effects free radicals may cause. It has been documented that DOX, an anthracycline type of chemotherapy, generated O_2_^•−^ [52], and antioxidants such as metformin alleviated DOX‐induced hepatic damages by attenuating the agent‐induced oxidative stress [53]. Similarly, cisplatin, a platinum‐based chemotherapeutic agent, induced acute kidney injury by inducing NOX‐mediated oxidative stress, and protocatechuic aldehyde (a naturally occurring phenolic aldehyde with antioxidative roles) attenuated such an adverse effect by suppressing the oxidative stress [54].

Industrial chemicals such as herbicides and pesticides also disrupt redox balance and induce oxidative stress (Figure 1A). For example, paraquat, a fast‐acting nonselective herbicide, induced the oxidative stress through generating O_2_^•−^ [55]. Paraquat‐induced oxidative stress has been implicated in neurodegenerative disorders such as Parkinson's disease, and natural antioxidants such as cryptotanshinone alleviated such syndromes via inhibiting ROS accumulation [56].

These findings have underscored the significant roles of free radicals in priming the cytotoxic effects of chemical agents. Understanding their mechanisms of action is crucial for developing strategies to enhance the efficacy while mitigating the adverse effects of these compounds for desirable outcome.

#### 2.2.3 Nanomaterials

Metal nanoparticles, such as titanium dioxide (TiO_2_) and silver nanoparticles, have gained significant attention due to their widespread applications in various industries including food, cosmetics, and medicine (Figure 1A). However, their potential to generate free radicals through mechanisms like surface plasmon resonance or Fenton‐like reactions has raised concerns regarding their safety and impact on human health. Surface plasmon resonance, a phenomenon where nanoparticles interact with light to generate localized electromagnetic fields, can produce ROS that can damage cellular components. Fenton‐like reactions, on the other hand, involve the reduction of H_2_O_2_ by metal ions to form highly reactive OH·. Both mechanisms contribute to the oxidative stress and can lead to various pathological conditions.

TiO_2_ nanoparticles, commonly used as food additives for whitening and opacification, have been shown capable of inducing colonic inflammation through ROS generation. A recent study demonstrated that TiO_2_ nanoparticles could trigger inflammation and induce fibrotic liver via modulating the level of 8‐hydroxydeoxyguanosine (8‐OHdG), and resveratrol (known antioxidant) reversed such a negative impact by attenuating the oxidative stress [57]. This may impose particular health concerns given the widespread use of metal nanoparticles in consumer products. Thus, carefully evaluating the long‐term safety of nanoparticle exposure and developing strategies accordingly are the key to mitigate their harmful effects while maintaining the full utilities.

#### 2.2.4 Environmental Factors

Environmental pollutants, including airborne particulate matter (PM2.5 and PM10), O_3_, and ultraviolet (UV) radiation, are significant contributors to the oxidative stress through the generation of free radicals (Figure 1A).

Fine particulate matter (PM2.5) is particularly hazardous due to its small size, which allows it to penetrate deep into the lungs and even enter the bloodstream. PM2.5 contains transition metals and polycyclic aromatic hydrocarbons that catalyze ROS production through Fenton reactions and redox cycling [58]. It has been reported that exposure to PM2.5 was associated with elevated mtROS in lung epithelial cells, driving inflammation via the activation of the NLRP3 inflammasome [59]. In addition, total PM2.5 includes several elements that may react with the hydrophilic environment, leading to a rapid release of bioactive water‐soluble compounds into circulation [60, 61]. It has also been demonstrated that aqueous PM2.5 promoted lipid accumulation and oxidative stress in the heart [62, 63, 64] and elicited a strong inflammatory response in alveolar epithelial cells [65].

O_3_ is another potent oxidant that reacts with pulmonary lipids and proteins to form lipid ozonides and aldehydes, decomposing into highly reactive species such as OH·. Chronic exposure to O_3_ has been shown to increase oxidative DNA damage, as evidenced by elevated levels of 8‐OHdG, a marker of DNA oxidation, in bronchial cells [66]; chronic O_3_ exposure was significantly associated with elevated hepatic fibrosis among rural women according to a cohort study (registration number: ChiCTR‐OOC‐15006699) [67]. Yet, O_3_ therapy was shown protective against acute lung injury and pulmonary fibrosis [68], suggesting the dual roles of free radicals played in human health and disease as well as the importance of dosage calibration.

UV radiation, particularly UV‐A (320–400 nm) and UV‐B (290–320 nm), penetrates the skin and generates ROS through the photosensitization of endogenous chromophores. UV‐A exposure has been shown to induce mtROS in human keratinocytes, leading to activated matrix metalloproteinase‐1 (MMP‐1) and accelerated skin photoaging [69]. Accordingly, hispidulin and sulforaphane showed promising antiphotoaging effects via suppressing MMP‐1 and collagen depletion [69]. Paradoxically, nanoparticles like zinc oxide (ZnO), commonly used in sunscreens for UV protection, have been shown effective in generating ROS and causing toxicity under UV light [70], raising safety concerns.

These findings have underscored the multifaceted mechanisms by which environmental factors contribute to various adverse health outcomes by inducing the generation of free radicals, understanding of which is crucial for developing strategies to mitigate these harmful effects for improved public health.

In summary, free radical generation stems from interconnected endogenous sources as well as diverse exogenous origins. Endogenously, oxidative phosphorylation during mitochondria respiration and pathological hyperglycemia represent major sources for free radical generation that underpin the centrality of O_2_ and metal ion in keeping the integrity of redox homeostasis. Exogenously, lifestyles such as tobacco smoking and excessive alcohol consumption, adoption of chemicals such as chemotherapies and products containing engineered nanomaterials like ZnO, and environmental stressors such as air pollution and industrial chemicals can further exacerbate the oxidative stress. While endogenous sources reflect metabolic vitality versus dysfunction, exogenous inputs represent environmental adaptability challenges, which altogether maintain a delicate equilibrium.

## 3 Molecular Mechanisms of Redox Signaling

Redox signaling operates through dynamic regulation of specific molecular pathways, where free radicals paradoxically serve dual roles as cytotoxic agents and essential second messengers. This section delineates key redox‐primed molecular mechanisms, including the multilayer homeostatic machinery, duality nature, redox switch, and consequential biological impacts, which together underpin the fine‐tuned yet potent redox communication system.

### 3.1 Homeostatic Machinery

**FIGURE 2:**
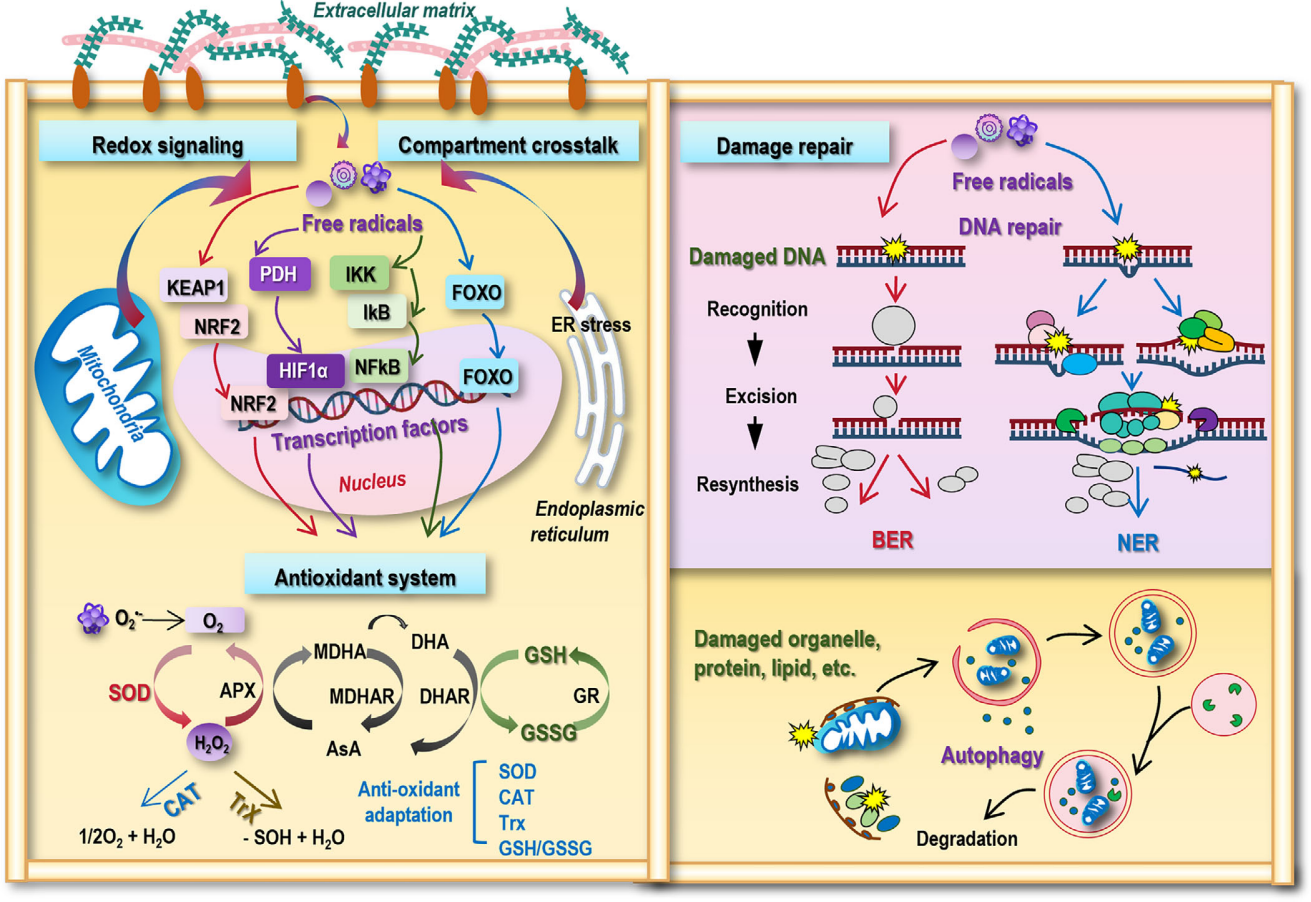
Mechanisms for maintaining redox homeostasis. The mechanisms for maintaining cells’ redox homeostasis are orchestrated at multiple levels that can be roughly classified as redox signaling, enzymatic and nonenzymatic antioxidant systems, compartment crosstalk, and damage repair. Cells have evolved adaptive metabolic programming such as dynamic fusion–fission cycles and metabolic mode switching to counteract oxidative stress. Endoplasmic reticulum (ER) can transfer disturbed proteostasis to ER stress. Enhanced stiffness and fibrosis of the extracellular matrix (ECM) can exacerbate the oxidative stress. These compartments dynamically crosstalk to pertain cellular redox homeostasis via initiating various redox signaling avalanche that are primarily mediated by transcription factors such as NRF2, NFκB, FOXO, and HIF1α. These signals stimulate the enzymatic and nonenzymatic antioxidant systems to take on appropriate adaptations in response to the redox stress. The antioxidant system is comprised of four major components, that is, superoxide dismutase (SOD), catalase (CAT), thioredoxin (Trx), and GSH systems, where SOD and CAT are enzymatic, and Trx and GSH are nonenzymatic systems. On the other hand, cells have evolved two primary repair mechanisms to avoid overproduction of free radicals. In the repair machinery, one mechanism is the DNA damage repair system that can be further grouped into base excision repair (BER) and nucleotide excision repair (NER); and the other mechanism is autophagy that serves as an important endogenous defense system against redox imbalance through degrading intracellular components.

Redox homeostasis is critical for cellular function, signaling, and survival. Disruption of this balance contributes to many pathological conditions that can be largely classified as degenerative and proliferative diseases. Sophisticated mechanisms have been employed by cells to maintain redox equilibrium, spanning redox signaling pathways, enzymatic and nonenzymatic antioxidant systems, repair and recycling mechanisms, as well as compartment crosstalk (Figure 2).

#### 3.1.1 Key Redox Signaling Pathways

Canonical pathways sensitive to redox perturbation include, primarily, signal transduction mediated by nuclear factor erythroid 2 related factor 2 (NRF2), forkhead box O (FOXO), NFκB, and hypoxia‐inducible factor 1 alpha (HIF1α) (Figure 2).

The transcription factor NRF2 regulates many antioxidant response elements inducing genes such as hemo oxygenase 1 and quinone oxidoreductase‐1 (NQO1). Fucoxanthin, a major marine carotenoid found in brown algae, has been shown capable of fighting against neuronal injury caused by amyloid‐beta (Aβ) accumulation via increasing the expression of NRF2 and its downstream genes encoding detoxifying enzymes such as NQO1 and thioredoxin reductase 1 (TrxR1) [71]. As another example, a small molecule activator compound 16 was characterized as a potential agent for inhibiting H_2_O_2_‐induced oxidative injury and death of osteoblasts through activating the NRF2 cascade [72]. In addition, the structural features from curcumin and diallyl sulfide have been integrated into nature‐inspired hybrid 1 in protecting retinal explants from the oxidative stress and neurodegeneration via stimulating the NRF2 pathway [8].

FOXO represent another family of transcription factors heavily involved in the regulation of antioxidant genes such as superoxide dismutase (SOD) and catalase (CAT) [73]. Oxidative stress‐responsive serine‐rich protein 1 (OSER1) was recently identified as an evolutionarily conserved protein regulated by FOXO; upregulated expression of OSER1 improved the antioxidant capacity of cells, leading to maintained mitochondrial functional integrity and prolonged lifespans of multiple species [74]. In consistent with this, the salivary level of FOXO1 was negatively correlated with that of 8‐OHdG, a marker of the oxidative stress, in periodontitis patients, suggesting the role of FOXO1 in supporting the antioxidant defense [75]. Voacangine, a naturally occurring alkaloid, has been proposed for treating ischemic stroke due to its demonstrated protective efficacy on hippocampal neuronal cells against oxidative stress by activating the FOXO cascade [76].

The NFκB pathway is a master regulator of inflammation, immunity, cell death and survival, which is critically modulated by cellular redox status. Inactive NFκB dimers are sequestered in the cytoplasm by inhibitor of NFκB (IκB). ROS can function as a key redox‐sensitive trigger of the IκB kinase (IKK) complex by directly activating IKKβ or inhibiting IκB phosphatase activity, leading to IκB phosphorylation and degradation, NFκB release and nuclear translocation, as well as activation of target genes encoding proinflammatory cytokines, chemokines, adhesion molecules, and antiapoptotic factors. In addition, ROS can act as second messengers in the NFκB pathway. For instance, mitochondrial or nitrogen oxides (NOX)‐derived ROS are required to fully activate IKK/NFκB signaling [77] and, conversely, sustained oxidative stress can inhibit NFκB DNA binding via oxidizing or nitrosylating Cys62 of NFκB [78].

The HIF1α pathway is a central hub in cellular adaptation to low O_2_, where ROS act as critical regulatory molecules. HIF1α is constitutively synthesized but rapidly degraded under normoxia. In this process, proline residues Pro402/Pro564 are hydroxylated by prolyl hydroxylase domain enzymes (PHD1‐3) at the expense of O_2_ and α‐ketoglutarate, and hydroxylated HIF1α is recognized by the von Hippel–Lindau E3 ubiquitin ligase complex that leads to proteasomal degradation. Crucially, PHDs are exquisitely sensitive to redox modulation. ROS can directly inhibit PHD activity through oxidizing ferrous iron (Fe^2+^) in the PHD catalytic center to ferric iron (Fe^3+^), or oxidizing critical cysteine residues. Consequently, ROS can help HIF1α escape degradation, leading to cytosol accumulation, nucleus translocation, dimerization with HIF1β, and transactivation of target genes involved in several critical cellular processes including, for example, angiogenesis (such as vascular endothelial growth factor), glycolysis (such as glucose transporter type 1, lactate dehydrogenase A), and erythropoiesis (such as erythropoietin). For instance, HIF1α‐mediated redox signaling regulated partial epithelial–mesenchymal transition (EMT) and mesenchymal–epithelial transition at primary and distant sites, respectively, during breast cancer metastasis [79]. HIF1α acted as a target of Ref‐1 (redox‐mediated transcriptional regulatory activity) during the regulation of retinal neovascularization [80]. Given these critical roles played by HIF1α in response to redox perturbation, HIF1α together with NRF2 have been proposed as emerging therapeutic targets for personalized redox medicine [81].

#### 3.1.2 Enzymatic and Nonenzymatic Antioxidant Systems

Cells possess several enzymatic antioxidants to neutralize overtly produced free radicals for redox homeostasis including, primarily, SOD and CAT [82, 83] (Figure 2). SOD enzymes catalyze the conversion of O_2_^•−^ to H_2_O_2_ and O_2_, which contain three isoforms, that is, cytosolic SOD1, mitochondrial SOD2, and extracellular SOD3. Nuclear presence of human SOD1 in the central nervous system (CNS) has been associated with the pathogenesis of amyotrophic lateral sclerosis according to an in vivo mouse model [84]. Hyper‐acetylation of the mitochondrial antioxidant SOD2 led to the pathogenesis of hypertension due to its loss‐of‐function in scavenging free radicals [85], possibly due to its mis‐cellular compartment localization. The importance of SOD2 in orchestrating redox homeostasis in intervertebral discs has also been systematically underscored [86]. SOD3^−/−^ mice demonstrated higher levels of 3‐nitrotyrosine, a key marker of oxidative stress, at the vitreoretinal interface, underlying the vital antioxidant role of SOD3 in preventing the pathology of diabetic vitreoretinopathy pathogenesis as well as other vitreoretinal diseases [87]. CAT degrades H_2_O_2_ to H_2_O and O_2_. Taking advantages of this feature, CAT‐like nanozyme‐hybrid hydrogels have been established to utilize endogenous free radicals as the O_2_ source to synergically regulate the oxidative stress for improved diabetic wound healing [88].

There are two major nonenzymatic antioxidant systems in cells, that is, Trx and GSH (Figure 2). The Trx system, which is comprised of thiorenitric oxidedoxin, TrxR, and NADPH, reduces disulfide bonds in oxidized proteins. The molecular characterization of TrxR from *Babesia gibsoni*, an erythrocytic intracellular parasite, was recently reported, the existence of which was considered to combat against the oxidative environment that these organisms are exposed to [89]. Thioredoxin‐2 is a small mitochondrial redox protein essential for the control of mtROS homeostasis, overexpression of which decreased the ROS level in rat embryonic ventricular myocytes [90] and alleviated the oxidative stress in bovine adipocytes via suppressing H_2_O_2_‐activated NFκB signaling [91]. GSH, the most abundant cellular thiol, directly scavenges ROS and serves as a cofactor for GSH peroxidase (GPX) and GSH‐S‐transferase. The GSH system can also be referred to as the GPX enzymatic system given the widely acknowledged role of GPX. GPX catalyzes the reduction of H_2_O_2_ and organic hydroperoxides (ROOH) to H_2_O or corresponding alcohols (ROH), utilizing reduced GSH as its essential electron donor, thereby protecting cells from oxidative damage through a catalytic cycle involving a selenocysteine (Sec) residue at its active site. The reaction is initiated when the peroxidatic selenol (Sec‐SeH) reduces the hydroperoxide substrate (H_2_O_2_/ROOH) to H_2_O/ROH, generating the selenenic acid intermediate (Sec‐SeOH); this oxidized intermediate then reacts with a molecule of GSH to form a selenylsulfide adduct (Sec‐Se‐SG); subsequently, a second GSH molecule attacks the selenylsulfide, regenerating the active selenol (Sec‐SeH) while producing oxidized GSH disulfide (GSSG); the resulting GSSG is then reduced back to GSH by NADPH‐dependent GSH reductase (GR), completing the antioxidant cycle [92]. This Sec‐dependent mechanism enables exceptionally efficient peroxide scavenging, with distinct GPX isozymes exhibiting substrate preferences. Specifically, GPX1 (cytosolic) primarily reduces H_2_O_2_ and soluble hydroperoxides, GPX2 (gastrointestinal) and GPX3 (extracellular) provide compartment‐specific protection, and GSH peroxidase 4 (GPX4) (phospholipid hydroperoxidase) uniquely reduces complex lipid hydroperoxides within membranes and also suppresses ferroptosis by inhibiting lipid peroxidation. The Sec residue, encoded by a UGA codon with a Sec insertion sequence element in the mRNA, is critical for catalysis due to the lower −log10 of the acid dissociation constant (i.e., p*K*_a_≈5.4) of selenium than cysteine thiols [93]. The involvement of the Sec residue in GPX ensures its high nucleophilicity at physiological pH for rapid peroxide reduction, and its involvement in the selenylsulfide intermediate prevents overoxidation to irreversible states like seleninic acid (Sec‐SeO_2_H) that allows sustained enzymatic activity under oxidative stress [94, 95]. By utilizing GSH to reduce lipid hydroperoxides and H_2_O_2_, GPX4 suppressed colorectal tumorigenesis through reducing manganese‐dependent oxidative stress [96]. In addition, glutamate–cysteine ligase (GCL), the rate‐limiting enzyme in GSH synthesis, was found declined with aging, the process of which is known being accompanied with enhanced oxidative status [97]. Accordingly, γ‐GC, the product of GCL and the immediate precursor of GSH, has been proposed with the therapeutic potential against aging due to its roles in bypassing impaired GCL and elevating cellular GSH [97].

#### 3.1.3 Damage Repair

DNA repair systems including the base excision repair (BER) and nucleotide excision repair (NER) machineries serve as the gatekeeper of the genome integrity via removing oxidative lesions and maintaining cells’ redox homeostasis (Figure 2). While BER corrects oxidative DNA lesions, NER addresses helix‐distorting oxidative damage. By administering a fluorescent probe to mitochondria that reacts rapidly with apurinic/apyrimidinic sites resulting from BER activity, elevated DNA repair activity under oxidative stress was observed, providing a direct linkage between BER activity and oxidative response [98]. CTC1–STN1–TEN1, a single‐stranded DNA binding protein involved in the maintenance of telomere length, was found capable of preventing the accumulation of oxidative DNA damage by stimulating the BER machinery [99]. Accumulating evidence has suggested that NER is also involved in neutralizing oxidative DNA damage besides BER. The role of NER proteins such as DNA damage recognition and repair factors A/C/G and cyclosporines A/B in the regulation of the cellular response to the oxidative damages has been underscored and considered as the underlying cause of many diseases including accelerated aging in Cockayne syndrome patients [100, 101].

Autophagy and mitophagy constitute as another repair mechanism for maintaining redox homeostasis (Figure 2). While autophagy removes oxidized proteins and organelles, mitophagy clears dysfunctional mitochondria, which is a key source of free radicals. Deficient autophagy has been associated with the progression of retinal degenerative diseases, since dysregulated retinal pigment epithelium cell proteostasis is accompanied by increased free radical generation and autophagy serves as an important endogenous defense system against redox imbalance through degrading intracellular components [102]. Codycepin alleviated metabolic dysfunction related liver disease by inducing Parkin‐mediated mitophagy that was associated with reduced oxidative stress and restored mitochondrial homeostasis [103].

#### 3.1.4 Compartment Crosstalk

Cells have evolved adaptive metabolic reprogramming to counteract oxidative stress, where mitochondria is heavily involved (Figure 2). On one hand, dynamic fusion–fission cycles segregate damaged mitochondria to prevent the spread of free radicals. On the other hand, cells may upregulate the pentose phosphate pathway (PPP) at the expense of mitochondrial respiration (as PPP provides an alternative route for glucose metabolism that diverts carbon flux away from glycolysis and the TCA cycle) to bypass the oxidative stress. A computational model of mitochondrial energy metabolism and free radical production has been established, underscoring the vital role of mitochondria played in redox homeostasis [104]. The beneficial impact of flavonoids on redox balance through regulating mitochondria dynamics has been systematically reviewed [105].

The endoplasmic reticulum (ER) is a central organelle involved in protein folding, lipid metabolism, and calcium homeostasis. In response to ER stress, the unfolded protein response (UPR) is activated to restore ER homeostasis (Figure 2). The UPR is initiated by three major ER transmembrane sensors, that is, protein kinase R‐like ER kinase (PERK), inositol‐requiring enzyme 1, and activating transcription factor 6 (ATF6). Upon ER stress, PERK is activated that phosphorylates eIF2α, leading to the selective translation of ATF4, a transcription factor that induces the expression of antioxidant genes; also, activated PERK promotes the stabilization of NRF2, one master regulator of the cellular antioxidant response [106].

The extracellular matrix (ECM) plays a significant role in redox regulation. Increased ECM stiffness and fibrosis can exacerbate the oxidative stress by activating pathways such as wingless‐related integration site signaling and transforming growth factor β (TGF‐β/SMAD); ROS‐activated TGF‐β1, in return, can lead to increased ECM production and fibrosis [107]. These pathways are interconnected with redox‐sensitive proteins to influence cellular redox states, highlighting the importance of maintaining ECM integrity in preventing chronic oxidative stress and tissue damage (Figure 2).

In all, this sophisticated homeostatic machinery comprised of redox signaling framework, enzymatic and nonenzymatic antioxidant systems, macromolecular damage repair mechanisms, and intercompartment crosstalk, determines whether free radicals serve as signaling messengers or drivers of oxidative injury that govern cellular resilience versus pathological decline.

### 3.2 Dual Roles

#### 3.2.1 Cellular Damaging

As a widely accepted conception, overt levels of free radicals deliver a multitude of negative impacts to cells by imposing the oxidative stress [108]. The oxidative stress, if not under appropriate control, is both a cause and an outcome of many disease syndromes covering both proliferative diseases such as cancers and degenerative syndromes such as neurological and cardiovascular disorder.

**FIGURE 3:**
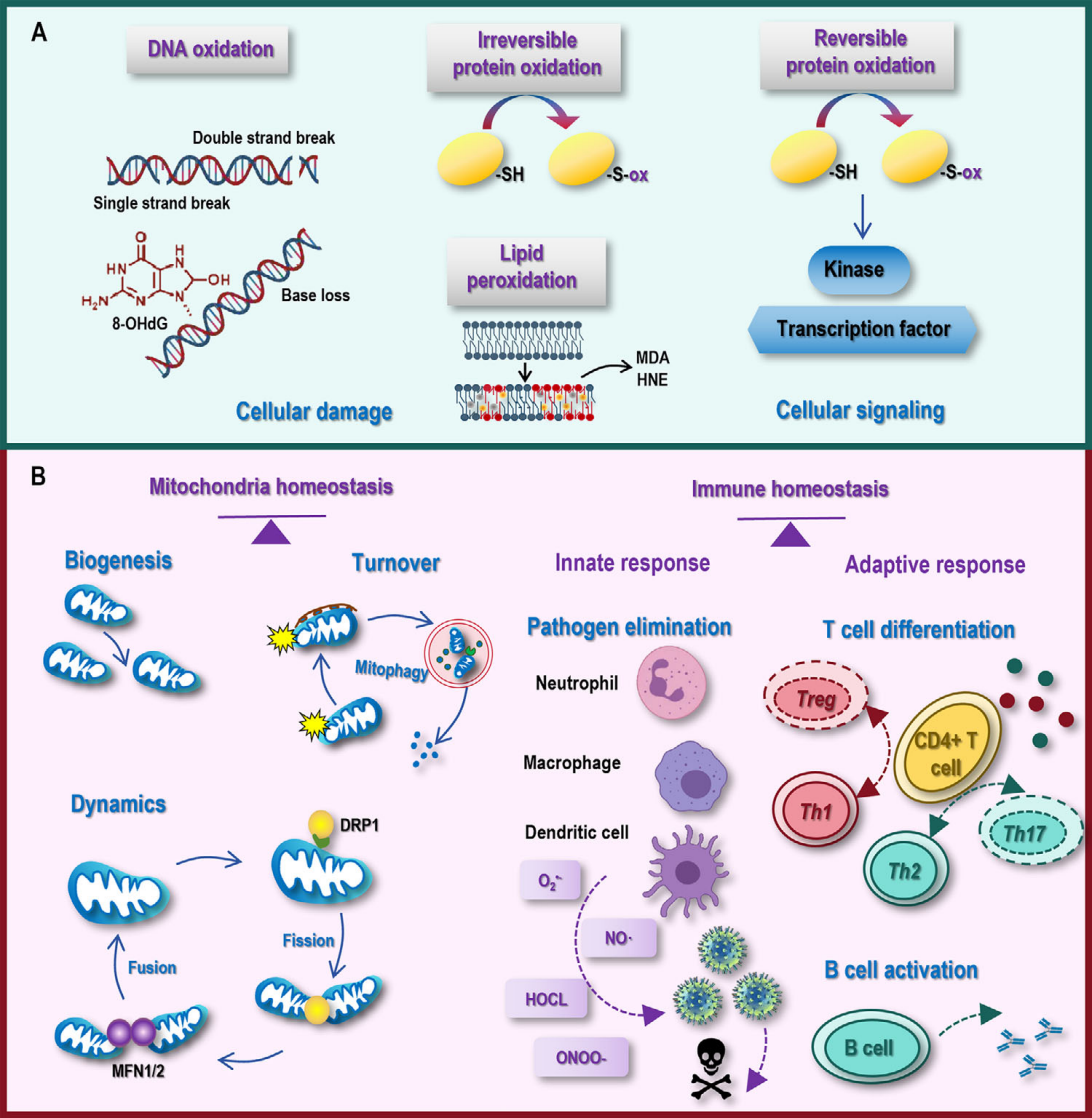
Dual roles and biological impacts of free radicals. (A) Free radicals have dual roles that can either create cellular damages to molecules such as DNA, protein, and lipids, or act as secondary messengers during cellular signaling. Among the varied types of short‐lived free radicals, OH· is the most reactive oxidant that forms adducts with all four DNA bases and reacts with multiple positions on ribose to cause the base loss and strand breaks. Out of the varied types of long‐lived species, H_2_O_2_ appears to be the major oxidation source that not only reacts with DNA but also generates CO_3_^·−^ to specifically target guanine oxidation and produce 8‐hydroxydeoxyguanosine (8‐OHdG), rendering DNA sequences in G‐quadruplex‐forming regions of gene promoters highly vulnerable to oxidative damages. Besides damaging DNA, excess free radicals can cause damages to proteins through irreversibly oxidizing cysteine residues, leading to dysfunctions of the affected protein, and induce lipid peroxidation by attacking polyunsaturated fatty acids in cell membranes that generate 4‐hydroxy‐2‐nonenal (HNE) and malondialdehyde (MDA) to further damage proteins and DNA. On the other hand, free radicals can affect cellular signaling through directly modifying specific cysteine residues in kinases and transcription factors or acting as secondary messengers to crosstalk with channel proteins and signal transduction molecules. (B) The primary beneficial impacts of free radicals to cells can be attributed to the maintenance of mitochondria homeostasis (existence) and immune homeostasis (protection). In mitochondria homeostasis, low to moderate levels of free radicals regulate mitochondrial dynamics, biogenesis and turnover toward mitochondrial homeostasis, and excessive free radical production can damage mitochondrial components. In immune homeostasis, phagocytes in the innate immune response such as neutrophils, macrophages and dendritic cells rely on free radicals to eliminate pathogens, where O_2_^•−^, HOCl, NO•, and ONOO^−^ are heavily involved. Immune cells in the adaptive immune system including T cells and B cells are critically regulated by free radicals regarding their differentiation and activation. Specifically, ROS can influence the differentiation of CD4^+^ T cells into T helper 1 (Th1), Th2, Th17, and T regulatory (Treg) cells, where Th1/Th2 and Th17/Treg form two critical pairs dictating the adaptive immunity. Maintaining adequate ROS production is essential during B cell activation as this process is subjected to an early oxidative step.

Free radicals are of the particular importance given their high activities in oxidizing the nucleic acids, where OH· is the most reactive oxidant reacting nonspecifically with biomolecules including DNA at diffusion‐controlled rates of *k*∼10^9^/M s) [109]. Take damages to the nucleic acids as the example, OH· forms adducts with all four DNA bases and reacts with multiple positions on ribose to cause the base loss and strand breaks. This leads to genetic mutations that, once accumulated and exceeded a certain threshold, may either transform cells into the pathogenesis state or initiate various death programs [108, 110] (Figure 3A). Though being transient, OH· can be generated through the reaction of certain transition metal ions (especially Fe^2+^ and Cu^+^) with H_2_O_2_, homolysis of H_2_O_2_, fission of H_2_O upon exposure to ionizing radiation, and homolysis of the excited water molecule (H_2_O)*, supporting its damaging activities in vivo [110] (Figure 3A). Although not a free radical itself, H_2_O_2_, a stably stored ROS often viewed as a product of free radical reactions, is the major source of nucleic acid oxidation, as it not only reacts with DNA but also generates ROS via reacting with Fe^2+^ [111, 112]. H_2_O_2_ can be produced either from O_2_^·−^ in mitochondria or be converted from O_2_ while performing a two‐electron oxidation on a substrate [113]. Alternatively, H_2_O_2_ can be generated from NO· and O_2_^·−^ during inflammation [114]. Recent advancements indicated that the primary damaging impact of H_2_O_2_ may not be itself, but rather the product it generates once reacting with carbon dioxide (CO_2_), that is, peroxynitrosocarbonate. Peroxynitrosocarbonate is an unstable compound that decomposes to NO_2_ and carbonate (CO_3_^·−^) through homolytic cleavage of the O‐O bond [115]. Unlike OH·, CO_3_^·−^ specifically targets guanine oxidation in DNA and RNA, resulting in 8‐OHdG that has epigenetic editing effects, especially in the context of a G‐quadruplex (Figure 3A). DNA sequences in G‐quadruplex‐forming regions of gene promoters are highly vulnerable to oxidative damage [108].

Excessive levels of free radicals can cause damages to proteins through oxidizing cysteine residues (Figure 3A). Cysteine residues predominantly exist as thiolate anions (Cys‐S^−^) under physiological conditions, which are highly reactive and can be oxidized to sulfenic acid (Cys‐SOH). This oxidation, once occurred, can alter the conformation and functionality of the affected protein. Such a process is reversible and a critical aspect of cellular redox signaling if the oxidative pressure did not exceed the tolerance threshold. However, excessive free radicals can push these modifications toward irreversibility, leading to cellular damage and dysfunction. For example, while nanomolar (nM) concentrations of H_2_O_2_ led to the onset of sulfenylation in living cells, higher levels of H_2_O_2_ led to permanent protein damage by forming sulfinic (SO_2_H) or sulfonic (SO_3_H) acids [116].

In addition, high oxidative levels can induce lipid peroxidation that is initiated when free radicals attack polyunsaturated fatty acids in cell membranes [116] (Figure 3A). This process begins with the abstraction of a hydrogen atom from a fatty acid, forming a lipid radical. The lipid radical then reacts with O_2_ to produce a peroxyl radical, which can further react with another fatty acid to propagate the chain reaction. This propagation leads to the formation of lipid hydroperoxides, which can decompose to form a variety of reactive aldehydes including 4‐hydroxy‐2‐nonenal (HNE) and malondialdehyde (MDA) [117, 118]. HNE and MDA have been used as markers of lipid peroxidation [117, 118]. Besides, they have biological activities that can affect cellular functions. HNE, for example, can modify proteins and DNA, leading to altered protein function and genotoxic effects. MDA, on the other hand, is a reactive aldehyde that can form adducts with proteins and DNA, leading to structural modifications and functional impairments.

#### 3.2.2 Cellular Signaling

Free radicals, particularly ROS and RNS, are no longer viewed solely as harmful byproducts of metabolism. Instead, they are recognized as critical regulators of cellular signaling pathways that govern processes ranging from growth and differentiation to apoptosis and immune responses (Figure 3A). These dual roles, that is, balancing physiological signaling and pathological damage, are regulated by their spatio‐temporal generation, concentration, and interaction with redox‐sensitive molecular targets.

Free radicals influence signaling through reversible oxidation of specific amino acid residues, particularly cysteine thiols, in proteins. They affect cell signaling through direct modification of signaling molecules and/or acting as secondary messengers. There are two ways that free radicals can directly edit signaling molecules, which are typically kinases and transcription factors (Figure 3A). In kinase activation, free radicals can oxidize cysteine residues in phosphatases, leading to inhibited kinase activity and prolonged signaling. For example, epidermal growth factor (EGF) stimulated NOX1 to generate H_2_O_2_, which inactivated polypyrimidine tract‐binding protein 1 B, leading to enhanced EGF receptor (EGFR)‐mediated cell proliferation [119]. In transcription factor modification, free radicals can modify the amount or activity of the transcription factors by oxidizing them or their regulatory proteins for altered protein stability. For instance, free radicals stimulated NFκB nuclear translocation and consequently the expression of proinflammatory genes by oxidizing its inhibitory IκB protein toward promoted degradation. Acting as the secondary messenger, ROS can crosstalk with channel proteins or interact with signal transduction molecules. For example, free radicals can activate the transient receptor potential membrane protein 2 channel, allowing for an influx of calcium (Ca^2+^) ions into cells that alters the intracellular calcium concentration [120, 121]. Also, NO• can bind to soluble guanylate cyclase (sGC) toward elevated cGMP levels, the signaling of which is involved in a number of important physiological processes such as smooth muscle relaxation and neurotransmission [122].

Several critical pathways have been implicated to be regulated by free radicals, including axes controlling cell proliferation such as the phosphatidylinositol 3 kinase (PI3K)/protein kinase B (AKT) pathway, cell death programs such as apoptosis, and inflammation such as NLRP3 inflammasome mediating signaling. For instance, H_2_O_2_ generated by NOX4 under glucose deprivation activated PI3K/AKT signaling in HepG2 liver cancer cells [123]. UV radiation‐induced mtROS activated the mitogen activated protein kinase (MAPK)/p38 axis, leading to the concomitant onset of apoptosis and NETosis (a unique form of neutrophil death) [124]. ROS potentiated persistent activation of NLRP3 inflammasome in microglia after whole‐brain radiation [125]; and mediated the pyroptosis of peripheral blood mononuclear cells via activating the NLRP3 inflammasome in Kawasaki disease [126].

### 3.3 Redox Switch

Central to balancing the dual roles played by free radicals in human health and disease is the redox switch, which is a sophisticated system that detects and responds to shifts in cellular redox states. Free radicals serve as dynamic redox sensors by modifying specific cysteine residues in proteins. These modifications alter protein conformation and activity, enabling cells to adapt to metabolic or environmental changes. For example, low levels of H_2_O_2_ oxidized cysteine thiols (‐SH) in kinases such as apoptosis signal regulating kinase 1 and phosphatases such as phosphatase and tensin homolog to adapt the growth and survival programs of cells in response to redox perturbation [127]; NO• edited FeS clusters and cysteine residues in proteins such as guanylate cyclase to influence vasodilation and neurotransmission that was subjected to a dynamic and rapid environmental regulation [128]. These redox‐sensitive modifications are reversible, allowing cells to reset their redox states once the stimulus subsides.

### 3.4 Biological Impacts of Free Radicals

#### 3.4.1 Mitochondrial Homeostasis

The role of free radicals in mitochondrial homeostasis is a complex and multifaceted topic that holds significant implications for cellular health and disease. Mitochondria, often referred to as the “powerhouses of the cell,” are responsible for generating the majority of the cell's energy in the form of ATP through oxidative phosphorylation. However, this process is accompanied by the production of free radicals, which can pose both beneficial and detrimental effects on mitochondrial homeostasis.

On one hand, low to moderate levels of free radicals play a crucial role in signaling pathways that regulate mitochondrial dynamics, biogenesis and turnover (Figure 3B). First, free radicals can act as signaling molecules that modulate mitochondrial fission and fusion events, ensuring the appropriate balance and morphology essential for efficient energy production. Specifically, mild ROS modulate mitochondrial dynamics by increasing the amount of fusion proteins such as mitochondrial fusion protein 2, and regulating the activity of fission proteins such as dynamin‐related protein 1, leading to minimized ROS leakage and the promotion of a connected mitochondrial network for efficient energy distribution [129]. Second, free radicals can participate in the regulation of mitochondrial biogenesis, the process by which new mitochondria are synthesized, thereby maintaining the mitochondrial network's integrity [130, 131]. In particular, low levels of ROS drive the expression of mitochondrial biogenesis‐related genes such as nuclear respiratory factor 1 (NRF1) and mitochondrial transcription factor A via activating AMP‐activated protein kinase (AMPK) and peroxisome proliferator‐activated receptor gamma (PPARγ) coactivator 1‐alpha (PGC‐1α) [131, 132]. Third, free radicals are involved in the regulation of mitophagy, keeping healthy mitochondria turnover [133, 134]. That is, low ROS can initiate mitophagy via mechanisms including, for example, recruiting Parkin E3 ubiquitin ligase to depolarized mitochondria [135, 136] and promoting the expression of mitophagy‐related genes such as microtubule‐associated protein 1 light chain 3 in pathways relying or not on sirtuin 1 (SIRT1), a NAD^+^‐dependent deacetylase [137, 138]. Thereby, maintaining free radicals within a hormetic zone may be feasible in treating pathological syndromes associated mitochondrial dysfunctions such as aging, neurodegeneration, and metabolic disorders.

On the other hand, excessive free radical production can lead to oxidative stress, a condition where the antioxidant defenses are overwhelmed, causing damages to mitochondrial components. These oxidative damages disrupt mitochondrial function, impair energy metabolism, and cause the release of proapoptotic signals, leading to a variety of pathological conditions. For instance, dysfunctional mitochondria as a result of mtROS overload in neurons overproduced O_2_^•−^ and OH· that oxidize mitochondrial DNA (mtDNA), disrupted ATP synthesis and calcium buffering, where metformin prevented this process via clearing mtROS [139]. Oxidized mtDNA leaked into the cytosol led to neurodegenerative syndromes such as Alzheimer's disease, and ginkgo biloba extract EGb761 ameliorated cell necroptosis by attenuating mtROS production [140]. In addition, ROS accumulation can cause age‐related metabolic syndromes by, for example, declining antioxidant defenses, inducing abnormal death of cells, and causing protein carbonylation. For example, mtROS accumulation has been associated with both obesity and type II diabetes by decreasing the expression of uncoupling protein 1 (a protein with antioxidative properties by regulating thermogenesis and energy balance) in beige adipocytes [141]; mtROS overproduction induced lipotoxicity on pancreatic β cells, leading to impaired insulin signaling and the pathogenesis of diabetes [142]; and mitochondrial O_2_^•−^ overproduction as a result of chronic high blood glucose exposure and AGE accumulation have been considered as one primary cause of diabetes and associated complications [143].

In summary, while free radicals are indispensable for normal mitochondrial function and signaling, their uncontrolled accumulation can disrupt mitochondrial homeostasis, contributing to a wide range of pathological conditions. Maintaining the integrate homeostasis between free radical production and antioxidant defenses is crucial for mitochondrial homeostasis and keeping human health.

#### 3.4.2 Immune Homeostasis

Immune cells, particularly phagocytes in the innate immune response such as neutrophils, macrophages and dendritic cells (DCs), rely on free radicals as critical tools to keep immune homeostasis for pathogen elimination and pathogenesis prevention (Figure 3B). Upon detecting pathogens via Toll‐like receptors (TLRs), neutrophils assemble NOX2 (the primary source of O_2_^•−^ in phagocytes) on phagosomal membranes that transfer electrons from NADPH to O_2_, leading to the generation of O_2_^•−^ in a rapid “respiratory burst”; neutrophils further amplify antimicrobial activity by converting O_2_^•−^ to hypochlorous acid (HOCl), where HOCl is exclusively associated with the innate immunity as a potent bactericidal/virucidal factor [144, 145]. For instance, inflammatory neutrophil‐derived HOCl has been found to critically contribute to the onset of ischemic angiogenesis [145]. Macrophages produce RNS such as NO• via iNOS during infections, which inhibit viral replication via modifying viral proteases. For instance, NO• originated from macrophages inhibited *avian influenza virus* by eliciting an antiviral immune response [146]. NO• reacts with O_2_^•−^ to form peroxynitrite (ONOO^−^), another toxic RNS capable of causing damages to cell membranes, proteins, and DNA, which can be used by immune cells for pathogen clearance. For example, macrophages used ONOO^−^ to damage Mycobacterium tuberculosis's DNA and lipids, limiting the replication of bacteria in tuberculosis [147]. The ability of DCs in cross‐presenting antigens to T cells is tightly regulated by the ROS level, with a low ROS level being considered necessary for them to take on normal activities. For instance, ROS derived from plasmacytoid DCs were shown protective against lupus [148]; and overt ROS accumulation dampened the functionality of DCs in presenting antigens to T cells [149].

Key players in the adaptive immune system including T cells and B cells are critically regulated by free radicals regarding their differentiation, activation, and proliferation [150] (Figure 3B). CD4^+^ T cells exhibit remarkable plasticity that can differentiate into various subsets such as T helper 1 (Th1), Th2, Th17, and T regulatory (Treg) cells, where Th1/Th2 and Th17/Treg form two critical pairs dictating the adaptive immunity under both physiological and pathological conditions [151]. ROS, acting as second messengers, can significantly influence the differentiation of CD4^+^ T cells. It has been shown that CD4^+^ cells deficient of the antioxidant gene *NRF2* led to Th1 polarization and increased production of proinflammatory cytokines [152, 153]. Paradoxical results have been documented on the role of ROS on Th17 differentiation. For instance, while the antioxidant N‐acetylcysteine (NAC) failed to prime Th17 differentiation in one study [154], opposite results were reported in another [155], suggesting the duel roles of free radicals played in T cell differentiation that need to be precisely controlled regarding the dosage to achieve the desirable outcome. In addition to affecting CD4^+^ T cell differentiation, ROS facilitate in the immuno‐suppressive functions of Treg cells. In support of this, eliminating ROS compromised Treg immunosuppressive functionalities by preventing SUMO‐specific peptidase 3 from ubiquitin‐mediated degradation [156]; and Tregs isolated from mice producing extremely low ROS due to loss‐of‐function of neutrophil cytosolic factor 1 (*Ncf1)* were hypo‐reactive [157]. CD8^+^ T cells require a fine tuning of ROS homeostasis to take on their activities. It has been shown that specifically depleting glutamate–cysteine ligase gene (*Gclc*) in CD8^+^ T cells resulted in significantly impaired inflammatory responses and less efficient T effector and memory differentiation during viral infection in vivo [158]. Maintaining adequate ROS production and redox balance is essential during B cell activation and differentiation. Naïve B cells are subjected to an early oxidative step triggered by B cell receptor (BCR) or TLR signaling, the process of which is associated with activated NOX1/3 followed by activated NFκB and PI3K signaling; and inhibiting NOX1/3 in B cells during BCR ligation displayed significantly decreased proliferation and failed immunoglobulin M antibody production [159, 160].

Given these aforementioned roles that free radicals play during immune innate and adaptive defenses, ROS act as fine‐tuners of the immune homeostasis, dysregulation of which contributes to symptoms such as immunosenescence, chronic inflammation and/or abnormal autoimmunity.

In summary, redox signaling exemplifies a context‐dependent molecular dialect. That is, while redox dysregulation drives cellular damage via oxidative stress, it is indispensable for cellular signaling under physiological conditions. Precision modulation through the redox switch orchestrates critical homeostasis in mitochondrial bioenergetics and immune responses. Deciphering this duality uncovers the key leading to the transition of healthy cells to various diseased forms.

## 4 Redox Dysregulation in Human Diseases

Redox dyshomeostasis, manifested as oxidative or reductive stress, constitutes a pleiotropic driver of human pathologies. This section examines the dichotomous roles of oxidative stress that fuels proliferative disorders and accelerates degenerative conditions, and underpins the criticality of reductive stress in priming disease onset, progression, and therapeutic resistance.

### 4.1 Oxidative Stress in Disease

#### 4.1.1 Oxidative Stress in Proliferative Disease

**FIGURE 4:**
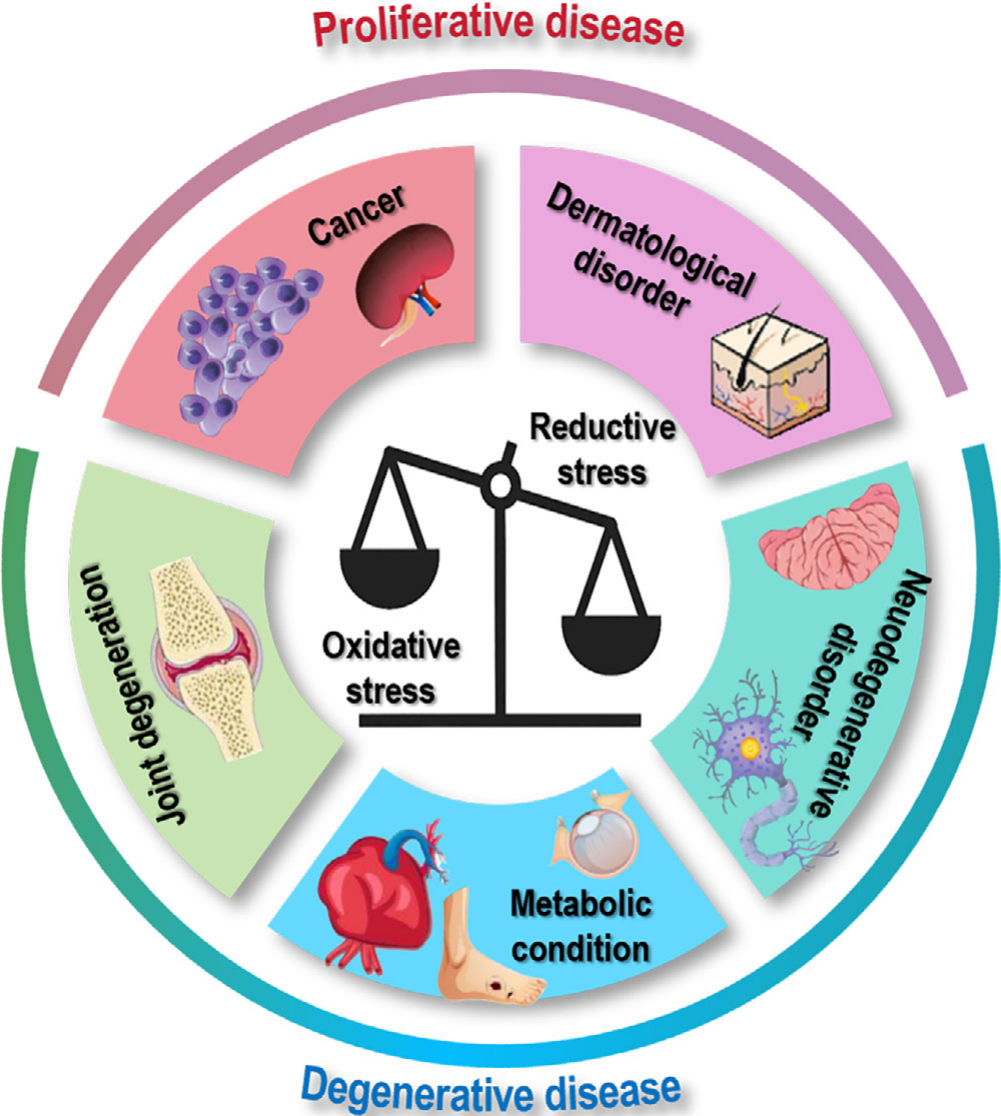
Pathological conditions caused by redox stress. Once cells are subjected to either oxidative or reductive stress, they may be primed to many pathological conditions. These include both proliferative diseases that are characteristic of uncontrolled cell growth such as cancers and some dermatological disorders, and degenerative diseases that are featured with progressive tissue damage and mitochondrial dysfunction such as neurodegenerative disorder, joint degeneration, and metabolic conditions. While the damaging effects of excessive oxidative stress in lipids, proteins, and DNA have been well recognized that drive the pathogenesis of many diseases, the roles of reductive stress in impairing redox‐sensitive signaling and promoting dysfunctions in proteins, lipids, and nucleic acids have often been overlooked.

While excessive free radicals can cause damages to cells, leading to genomic instability that drives the initiation and progression of many proliferative pathological conditions such as cancers and hyper‐proliferative skin disorders (Figure 4 and Table 1).

**TABLE 1:** Exemplified free radical based modalities for disease management.

Agent	Agent type	Agent efficacy	Disease	Disease type	Disease pathogenic mechanism	References
CAP	Antioxidant	Therapeutics	Rheumatoid arthritis	Degenerative	Kill fibroblast‐like synoviocytes and stimulate the self‐antioxidant capability of the surrounding healthy tissue	[272, 273, 274, 275]
CAP	Antioxidant	Therapeutics	Diabetes, diabetic foot	Degenerative	Reduce blood glucose level and boost tissue regeneration	[276, 277, 278, 279, 280]
Coenzyme Q10	Antioxidant	Therapeutics	Chronic ischemic myocardium	Degenerative	Improve mitochondrial homeostasis	[253]
Bardoxolone methyl	Antioxidant	Therapeutics	Diabetic neuropathy	Degenerative	Improve mitochondrial homeostasis by activating NRF2	[265, 266]
Mediterranean diet	Antioxidant	Prevention	Various diseases	Degenerative, proliferative	Reduce oxidative stress	[247, 248, 249]
Mitoquinone	Antioxidant	Prevention	Various diseases	Degenerative, proliferative	Protect corneal endothelial cells from oxidative damage and improve mitochondrial homeostasis	[251, 252]
Nicotinamide riboside	Antioxidant	Prevention	Cardiac complications	Degenerative	Improve mitochondrial homeostasis by activating SIRT	[270, 271]
Physical exercise	Antioxidant	Prevention	Alzheimer's disease, Parkinson's disease	Degenerative	Increase the expression of antioxidant enzymes and thus reduce inflammation via reducing oxidative stress	[254]
Resveratrol	Antioxidant	Therapeutics, prevention	Alzheimer's disease, Parkinson's disease	Degenerative	Improve mitochondrial homeostasis by activating SIRT	[268]
Sulforaphane	Antioxidant	Therapeutics	Alzheimer's disease, Parkinson's disease	Degenerative	Improve mitochondrial homeostasis by activating NRF2	[259, 260, 261, 262, 263]
CAP	Prooxidant	Therapeutics, drug sensitizer	Various cancers	Proliferative	Target all cancer hallmarks and sensitize cancer cells to existing therapies such as immunotherapy and chemotherapy by enhancing cellular oxidative stress	[225, 226, 227, 228, 229, 230, 231, 232, 233, 234, 235, 236, 237]
CAP	Prooxidant	Therapeutics	Psoriasis, keloid	Proliferative	Inhibit hyper‐proliferation of keratinocytes and attenuate abnormal inflammation for restored cytokine homeostasis	[240, 241, 242, 243, 244]
CAP	Prooxidant	Therapeutics	Malassezia folliculitis, herpes zoster	Proliferative	Kill pathogens by enhancing cellular oxidative stress	[245, 246]
Auranofin	Prooxidant	Therapeutics	Advanced cancers	Proliferative	Kill cancer stem cells by enhancing mitochondria oxidative stress	[214]
Brusatol	Prooxidant	Therapeutics, drug sensitizer	Non‐small cell lung cancer, bladder cancer, thyroid cancer, pancreatic cancer	Proliferative	Kill cancer cells or sensitize cancer cells to prooxidant agents by enhancing cellular oxidative stress	[217, 218, 219, 220, 221]
Doxorubicin	Prooxidant	Therapeutics	Lung cancer	Proliferative	Kill cancer cells by enhancing cellular oxidative stress	[216]
Resveratrol + copper	Prooxidant	Toxicity reducer	Gastric cancer	Proliferative	Deactivate cell‐free chromatin particles released from chemotherapy‐induced dying cells via generating oxygen radicals	[212]
Sorafenib	Prooxidant	Therapeutics	Liver fibrosis, renal fibrosis	Proliferative	Trigger hepatic stellate cell ferroptosis by enhancing cellular oxidative stress	[222, 223, 224]

The oxidative stress is integral to the hallmarks of cancer [161] that spans disease initiation and progression, acting as double‐edged swords in tumor biology. While low levels of free radicals function as signaling molecules to modulate pathways critical for cell proliferation and survival, chronic oxidative stress induces genomic instability that creates a permissive environment for clonal expansion, leading to cell transformation. Cancer cells exploit elevated ROS to sustain uncontrolled growth, evade programmed death events, enable replicate immortality, promote abnormal angiogenesis, stimulate metastasis, rewire the metabolism, evade immune surveillance, and enhance chronic inflammation. For instance, oxidative stress related genes such as Kelch‐1 like ECH‐associated protein 1 (*Keap1*) and *NRF2* have been reported contributing to the risk of developing epithelial ovarian cancers [162], and the roles of redox stress in priming breast cancers have been particularly emphasized [163]. Individual oxidative stress markers have been used to assess the chance of developing colorectal cancer liver metastasis, with a poor outcome being associated with relatively high redox stress scores [164]. Therapeutically, a 6‐n‐butoxy‐10‐nitro‐12,13‐dioxa‐11‐azatricyclo[7.3.1.0^2,7^]trideca‐2,4,6,10‐tetraene (namely SK2), synthesized based on the naturally existing benzo‐fused dioxabicyclo[3.3.1]nonane skeleton [165], suppressed the proliferation of oral cancer cells with minimal damages to normal cells [166] and sensitized cancer cells to X‐ray irradiation by enhancing cellular ROS [167]. An oxidative stress nanoamplifier has been developed to enhance the treatment efficacy of cisplatin in resolving head and neck cancers [168].

The oxidative stress has been considered as a primary cause of dermatological disorders with hyper‐proliferative features such as psoriasis. In psoriasis, ROS in psoriatic keratinocytes hyperactivate interleukin (IL)‐23/IL‐17 signalings to foster uncontrolled proliferation, and mtROS perpetuate epidermal thickening by enhancing immune cell infiltration. DNA nanostructures incorporated into microneedles capable of delivering IL‐17A siRNA into psoriatic lesions were fabricated that showed a great promise in treating psoriasis by dampening ROS levels [23]. Additionally, combined use of monomethyl fumarate and aluminum ions showed a superior efficacy in treating psoriasis in vivo via modulating the NRF2/NFκB pathway toward diminished oxidative stress [169]. As another evidence supporting the driving role of the oxidative stress on the pathogenesis of psoriasis is the clinical pattern of pustular psoriasis of pregnancy. The condition of this particular subtype of psoriasis typically exacerbates during advanced stages of gestation and generally resolves following childbirth, with a demonstrated strong propensity for recurrence in future pregnancies. Such a dynamic pathological pattern coincides with the oxidative state of the patients, where the oxidative stress is physiologically elevated during the pregnancy as a result of intensified mitochondrial activity in placental tissues and heightened free radical generation required to support fetal development [7]. Infliximab, an anti‐NFκB antibody, has been shown effective in treating a 22‐year‐old woman carrying generalized pustular psoriasis of pregnancy without detected serious adverse events [170]. Similarly, the antibody against IL‐17, that is, secukinumab, also displayed remarkable therapeutic outcomes in treating psoriasis including this particular subtype [171].

#### 4.1.2 Oxidative Stress in Degenerative Disease

Oxidative stress is a central driver of many degenerative diseases such as neurodegeneration, joint degenerative diseases, and metabolic syndromes by causing progressive tissue damage and mitochondrial dysfunction (Figure 4 and Table 1). This is because that excessive ROS can damage lipids, proteins, and DNA, leading to disrupted cellular signaling and accelerated cell death.

Many neurodegeneration conditions such as Alzheimer's disease and Parkinson's disease may be afflicted by the oxidative stress. In Alzheimer's disease, Aβ aggregates interact with metal ions such as Fe^2^^+^ and Cu^+^ to generate ROS and catalyze the Fenton reaction. The resulting ROS oxidize lipids in neuronal membranes, leading to impaired synaptic function. Concurrently, oxidative stress activates kinases such as glycogen synthase kinase 3β, which phosphorylate tau protein and promote neurofibrillary tangle formation. Furthermore, Aβ accumulation in the mitochondria inhibits complex IV (i.e., cytochrome *c* oxidase), increasing ROS production and establishing a vicious cycle of oxidative damage [172]. A progressive age‐related accumulation of oxidative damage to DNA was detected in the human brain where mtDNA was preferentially affected. Rapidly progressive Alzheimer's disease showed exacerbated mitochondrial dynamic abnormalities without evident tau pathology, underscoring the high sensitivity of mitochondria to the oxidative stress [24]. Experimentally, the amount of 8‐OHdG was elevated in the neurons of individuals carrying the Alzheimer's disease [24, 173]; 8‐OHdG was elevated in patients suffering from severe mental illness [174]. The Parkinson's disease is characterized by a gradual loss of dopaminergic neurons as a result of dopamine oxidation that leads to pathophysiological changes in the downstream basal ganglia circuit and subsequent motor dysfunction [175]. In addition, oxidative stress aggregates mitochondria damages by impairing the mitophagy of abnormal mitochondria. Recent advancements have underscored the critical roles played by long noncoding RNAs (lncRNAs) in damages caused by the oxidative stress during the pathogenesis of Parkinson's disease [176]. For example, 1‐methyl‐4‐phenyl‐1,2,3,6‐tetrahydropyridine is neurotoxic that can selectively damage neuron once oxidized to its active form, that is, MPP+ [177]; suppressing the expression of the lncRNA taurine‐upregulated gene 1 (*TUG1*) alleviated neuro‐inflammation by reducing tumor necrosis factor α (TNFα) and IL‐1β expression in MPP+‐induced cells, and miR‐152‐3p decreased pathological damages caused by ROS in the substantia nigra of Parkinson's disease in vivo by sponging *TUG1* [178]. As another example, upregulated expression of *NORAD* (lncRNA activated by DNA damage) alleviated MPP+‐induced apoptosis and mitochondrial dysfunction by reducing ROS activity in an in vitro model of Parkinson's disease [179].

Oxidative stress plays a crucial role in the development of joint degenerative diseases such as osteoarthritis and intervertebral disc degeneration. Osteoarthritis is a degenerative joint disorder characterized by progressive cartilage degradation, synovial inflammation, and subchondral bone remodeling. The pathogenesis of osteoarthritis involves an imbalance between cartilage matrix synthesis and catabolism, where the oxidative stress plays an indispensable role [180]. For instance, ethylene oxide exposure has been associated with enhanced osteoarthritis risk, and it was the oxidative stress instead of inflammation that built up such a strong correlation according to a National Health and Nutrition Examination Survey from 2013 to 2020 [181]. In addition, the redox homeostasis of osteoarthritis patients was disrupted by free radicals, which led to increased inflammation, decreased immunity, and accelerated disease progression; and alterations on parameters such as total antioxidants, oxidative stress, and thiol‐disulfide balance values were in direct proportions to disease staging, especially among patients having already progressed into stages III–IV [182]. In intervertebral disc degeneration, oxidative stress causes the breakdown of ECM components in the intervertebral disc, resulting in reduced shock‐absorbing capacity, disc degeneration, and spinal instability [183]. Melatonin has been shown protective against intervertebral disc degeneration by inhibiting the oxidative stress, leading to inhibited matrix degeneration enzymes such as MMP‐13 and preserved ECM contents such as SRY‐box transcription factor 9, aggrecan, and collagen II [183]. The antioxidant naturally present in a variety of plants, gallic acid, has been shown effective in alleviating intervertebral disc degeneration via attenuating the ferroptosis of nucleus pulposus cells and reducing ECM degradation [184]. Accordingly, gallic acid derived PGA–Cu nanoparticles enhanced with functional octapeptide (Cys–Lys–His–Gly‐d–Arg‐d–Tyr–Lys–Phe, SS08) was fabricated and embedded within a hydrogel matrix for treating intervertebral disc degeneration, and such a nanocomposite hydrogel exhibited excellent biosafety and treatment performance through sustained drug release [185].

Oxidative stress is a critical contributor to the development and progression of metabolic conditions such as atherosclerosis, hypertension, myocardial infarction, obesity, type 2 diabetes, and nonalcoholic fatty liver disease by disrupting metabolic homeostasis and accelerating tissue injury. Atherosclerosis is a chronic immune inflammatory disease due to excessive free radical production. Mechanically, ROS can trigger the apoptosis of endothelial cells and proinflammatory vascular responses, leading to the infiltration of immune cells and inflammatory cytokines into the vascular wall and, consequently, the onset of atherosclerosis [186]. Inflammatory cytokines such as TNF and IL‐1 activates the NFκB pathway, contributing to increased ROS generation, and the mutual promotion between inflammation and oxidative stress plays an indispensable role during the initiation and development of atherosclerosis [186]. Experimentally, upregulation of oxidative stress‐related genes such as isocitrate dehydrogenase 1 and cluster of differentiation 36 in macrophages was found critical in driving the formation and progression of carotid atherosclerotic plaques [186]. Monotropein, an antioxidant compound found in herbaceous plants *Morindae officinalis*, inhibited the proliferation and migration of vascular smooth muscle cells by reducing the oxidative stress and thus was proposed as a protective agent against atherosclerosis [187]. Hypertension is characterized by impaired endothelial functionality and vascular inflammation that is closely linked to the oxidative stress. By activating proinflammatory pathways and the renin–angiotensin–aldosterone system, oxidative stress amplifies angiotensin II‐induced vasoconstriction and ROS production, perpetuating vascular remodeling, arterial stiffness, and renal sodium retention [188]. Antioxidant therapy offers the potential to mitigate the detrimental effects of hypertension that may inflict various organs by alleviating the oxidative stress. For instance, polyphenols, found in many plant‐based foods, showed promise in managing hypertension. An inverse and nonlinear association was identified between dietary polyphenol intake (especially lignan and stilbene) and the risk of developing hypertension [189]. Hypertension associated complications such as erectile dysfunction may also be cured by alleviating the oxidative stress, where suppressing NLRP3 inflammasome‐dependent endothelial cell pyroptosis via activating the NRF2/HO‐1 axis was proposed as a promising therapeutic strategy [190]. The pathogenesis of myocardial infarction involves a complex interplay between ischemic injury and oxidative stress. Following coronary artery occlusion, myocardial ischemia disrupts mitochondrial electron transport, leading to excessive ROS generation; this oxidative surge overwhelms endogenous antioxidant defenses, resulting in damages to endothelial cells, impaired cardiomyocyte contractility, and exacerbated inflammation [191]. It has been shown that hyperbaric O_2_ preconditioning protected against acute myocardial infarction injury through macrophage stimulating 1‐mediated Keap1/NRF2/HO‐1‐dependent antioxidant defense system [192]. Ciliary neurotrophic factor prevented cardiac hypertrophy and cardiac fibrosis via initiating the PI3K/Akt axis that led to reduced oxidative stress and ferroptosis in response to myocardial infarction damage [193]. Incremental evidence has associated obesity with the oxidative stress. Excess adiposity leads to increased ROS generation through triggering mitochondrial dysfunction and inflammation. Adipose tissue in obesity undergoes hypoxia due to rapid expansion that exacerbates the oxidative stress. This oxidative imbalance damages cellular components and disrupts adipokine secretion, leading to impaired energy homeostasis and weight gain. Experimentally, the marker indicating the redox status, that is, GSH/GSSG ratio, and molecules indicating protein carbonylation, that is, IL‐6 and S100B (S100 calcium binding protein B) were higher in elderly obese Mexican women carrying cognitive impairment as compared with the control [194]. The interplay between obesity and oxidative stress creates a vicious cycle that accelerates metabolic complications and highlights the need for therapeutic strategies targeting oxidative pathways. As an example, the consumption of cashew nuts was found to reduce the waist circumference of obese individuals by enhancing the antioxidant defense [195]. Two bioactive peptides PIISVYWK (P1) and FSVVPSPK (P2), derived from the blue mussel *Mytilus edulis*, exhibited significant benefits in combating against obesity via alleviating the oxidative stress and inflammation. Specifically, these peptides inhibited the differentiation of bone marrow‐derived mesenchymal stem cells into adipocytes by downregulating PPARγ, CCAAT/enhancer‐binding protein alpha, and sterol regulatory element‐binding protein 1, reduced lipogenesis and enhanced lipolysis via activating AMPK and hormone‐sensitive lipase [196]. The pathogenesis of type 2 diabetes is marked by excessive free radical generation that overwhelms the antioxidant defense. Specifically, chronic hyperglycemia and hyperlipidemia drive ROS generation as a result of mitochondrial dysfunction and AGE production. ROS cause damages to pancreatic β cells and impair insulin signaling toward reduced insulin secretion and promoted insulin resistance. These interconnected mechanisms drive the progression of type 2 diabetes and its metabolic derangement [197]. Strategies targeting the oxidative stress have been proposed for treating type 2 diabetes. Preclinically, fruit extracts of different cultivars of the cornelian cherry alleviated hyperglycemia and the oxidative stress in a rat diabetes model [198]. Salusin‐α mitigated the symptoms of gestational diabetes in an in vivo model by alleviating the ROS stress in the placental tissue [199]. In the clinics, supplementation of the antioxidant resveratrol improved the oxidative stress and disease symptoms in type 2 diabetes patients according to a randomized double‐blind placebo trial involving 533 participants [200]. Nonalcoholic fatty liver disease is featured by excessive lipid accumulation, especially triglycerides, within hepatocytes; and this is hypothesized to make the liver susceptible to the oxidative stress [201]. It has been shown that the oxidative stress can drive the progression of nonalcoholic fatty liver disease (NAFLD) to nonalcoholic steatohepatitis with fibrosis, where enhanced levels of oxidative stress markers were evident in these patients [202]. Therapeutically, ellagic acid has been proposed as a supplemental therapy together with existing treatment modalities to reduce the complications of nonalcoholic fatty liver disease due to its antioxidant properties [203]. Anthocyanins, natural flavonoids with known antioxidative activities, have been shown to be beneficial to liver health [204]. The value of using antioxidants from food in reducing the incidence of NAFLD has been evaluated in a National Health and Nutrition Examination Survey from 2005 to 2016, and the results showed a negative association between antioxidant intake and disease onset in US adult population that highlighted the potential for dietary intervention to reduce the disease incidence [205].

### 4.2 Reductive Stress in Disease

Reductive stress, an often‐overlooked counterpart to oxidative stress, refers to a pathological state characterized by an excessive accumulation of reducing equivalents or hyperactivation of antioxidant systems, disrupting the delicate balance of cellular redox homeostasis (Figure 4 and Table 1). Unlike oxidative stress, where ROS overwhelm antioxidant defenses, reductive stress arises when the cellular environment becomes overly reduced, impairing redox‐sensitive signaling and promoting dysfunction in proteins, lipids, and nucleic acids.

Reductive stress encompasses three distinct metabolic stress states, which are NADH‐reductive stress as a result of imbalanced NADH/NAD+ ratio, NADPH‐reductive stress as a result of imbalanced NADPH/NADP+ ratio, and GSH‐reductive stress as a result of imbalanced GSH/GSSG [206]. NADH‐reductive stress occurs when there is an imbalance in the NADH/NAD+ ratio, typically due to excessive production of NADH or impaired oxidation pathways. NADH is a key electron carrier in cellular respiration, and its accumulation can disrupt energy production and lead to oxidative stress paradoxically, since excess NADH can overwhelm the ETC that leads to free radical generation. NADPH‐reductive stress involves an imbalance in the NADPH/NADP+ ratio, often resulting from increased NADPH production or reduced consumption. NADPH plays a critical role in biosynthetic reactions and the maintenance of redox balance. An excess of NADPH can lead to lipid peroxidation and other forms of cellular damage if not properly managed by metabolic pathways. GSH‐reductive stress arises from an imbalance in the GSH/GSSG ratio, where the level of the reduced form of GSH is disproportionately high than that of the oxidized form (GSSG). GSH is essential for detoxifying reactive species and maintaining protein thiol redox states. However, excessive GSH can interfere with normal signaling processes and contribute to pathological conditions if not balanced appropriately.

Over the past three decades, studies on redox biology have been centered around the concept of “oxidative stress.” However, it has been now gaining incremental recognition on the potential dangers that the opposite end of the cellular redox spectrum, that is, reductive stress, may impose to human health. An overly reductive environment as a result of disrupted redox imbalance is detrimental to cells, which can be linked to various diseases including proliferative syndromes such as cancers and degenerative diseases such as neurodegenerative disorders [206]. Take the GSH/GSSG axis as the example, excessive NADPH production via, for example, PPP hyperactivity, may elevate reduced the GSH level that overwhelms the GSSG recycling capacity, leading to an abnormally high GSH/GSSG ratio and disrupted redox‐sensitive signaling. The reductive stress may occur in hypoxia and also in several diseases where a small but persistent generation of oxidants results in a hormetic overexpression of antioxidant enzymes that leads to a reduction in cell compartments.

Reductive stress creates an overly reduced cellular environment that endangers the genome integrity and disrupts central biochemical pathways, driving the progression of various diseases. In addition, transformed cells may upregulate the antioxidant systems to mitigate ROS toxicity and develop resistance to many existing therapies, highlighting free radicals as central mediators of cancers’ complexity and adaptability. Understanding these mechanisms may provide insights into potential therapeutic targets and strategies to mitigate associated health issues. In support of this, relatively high levels of GSH were found to be associated with enhanced chemo‐resistance in many types of cancers [207]; and overactivity of NRF2 was linked with many types of malignancies and poor clinical prognosis [208]. Therapeutically, a core–shell nanostructure of cadmium telluride quantum dots with mesoporous silica coating was fabricated and functionalized with poly(2‐vinylpyridine)‐polyethylene glycol‐folic acid, and this nanostructure triggered the apoptosis of liver cancer cells through inducing the reductive stress under hypoxia [209]; adjuvants capable of inducing the reductive stress such as ellagic acid improved the therapeutic effectiveness of radiation therapy under insufficient O_2_ supply [206, 210].

In addition, monitoring these ratios and understanding their dynamics may offer valuable clues on cellular health and response to stressors for early disease prevention. Though the oxidative stress has been widely accepted as the primary force driving the progression of degenerative diseases, individuals at a high risk of developing degenerative disorders may suffer from the reductive stress long prior to the onset of the syndromes. For example, reductive stress has been found in the animal model of the Alzheimer's disease (i.e., the *APP/PS1* transgenic mice), where the redox state of mice was determined at their young age before the onset of the disease [211]. Thus, characterizing the reductive stress prior to any signs or symptoms of degenerative diseases is of the critical importance as it may serve as a very early marker for disease prevention.

In summary, both oxidative and reductive stress may disrupt the redox homeostasis, which synergistically drive the transition from physiological equilibrium to pathological states. While oxidative stress promotes pathological proliferation and degeneration via causing macromolecular damages, reductive stress disrupts redox signaling fidelity through electron overflow. Deciphering this duality, particularly cell‐type‐specific stress signatures, is critical for developing targeted redox‐modulating therapies.

## 5 Redox Therapeutic Targeting Strategies

Therapeutic redox interventions demand pathology‐stratified precision. That is, while prooxidant strategies exploit elevated ROS in proliferative diseases such as cancers to induce selective cytotoxicity, antioxidant approaches counteract oxidative damage in degenerative disorders. This section evaluates conventional agents alongside CAP, a tunable modality adaptable to both paradigms.

### 5.1 Prooxidant Strategies for Proliferative Disease

**FIGURE 5:**
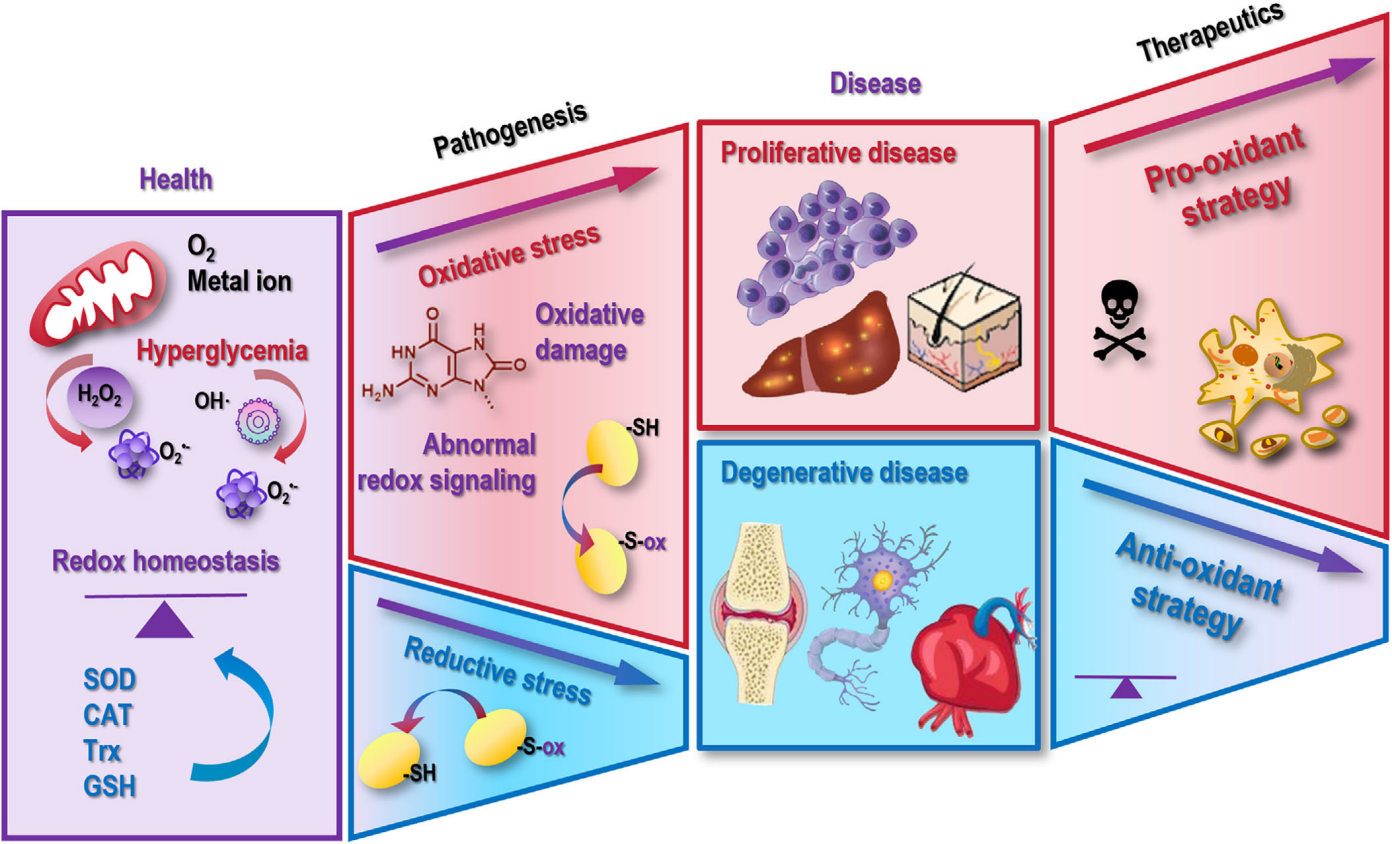
Therapeutic approaches relying on redox regulation. When cells are attracted in the “Health” state, cellular redox homeostasis is maintained by the balance between the generation source of free radicals and the antioxidant systems. The primary endogenous source for free radical generation is mitochondrial respiration, where leaky electrons from the electron transport chain (ETC) interact with O_2_ to produce O_2_^•−^ or H_2_O_2._ Hyperglycemia offers a central condition for generating endogenous free radicals such as OH· and O_2_^•−^, with glycation and glucose autoxidation being the primary events that may both involve transition metals such as iron (Fe^2+^) and copper (Cu^+^) through the Fenton and Haber–Weiss reactions. These emphasize the centrality of O_2_ and transition metal in free radical generation and redox homeostasis. Cells adopt four major systems to neutralize intracellular free radicals that are superoxide dismutase (SOD), catalase (CAT), the thioredoxin (Trx) system, and the glutathione (GSH) couple. During pathogenesis, the redox homeostasis is disrupted, leading to either oxidative stress or reductive stress. While the oxidative stress can cause oxidative damages to molecules such as DNA and trigger abnormal redox signaling by inducing oxidization of specific amino acid residues especially cysteine thiols, the reductive stress can disturb normal redox signaling by reducing oxidized cysteine thiols. When cells enter the “Disease” state, they either develop syndromes characteristic of uncontrolled cell growth such as cancers and some dermatological disorders or evolve into symptoms featured with progressive tissue damage and mitochondrial dysfunction such as neurodegenerative disorder, joint degeneration, and metabolic conditions. Therapeutically, pro‐oxidative approaches further enhance the redox level of cells characteristic of uncontrolled rapid proliferation to exceed their death threshold, leading to specific death of pathologic cells given their relatively higher redox level than the healthy cells; antioxidant approaches neutralize excessive free radicals to restore cellular redox homeostasis that are typically applied for treating diseases characteristic of progressive tissue damage.

Prooxidant therapies have evolved as a promising approach in the treatment of proliferative conditions such as cancers and some dermatological diseases by selectively targeting and killing diseased cells. These strategies exploit the inherent oxidative stress in rapidly proliferating cells, enhancing their vulnerability to ROS‐mediated damages. This elevated oxidative stress is due to the increased metabolic activity of rapid proliferating cells, which generates higher levels of free radicals. By further increasing the ROS level, prooxidant therapies can push cancer cells beyond their capacity to neutralize the oxidative damage, leading to various forms of programmed death events. Additionally, prooxidant strategies can enhance the sensitivity of diseased cells to existing treatments such as chemo‐ and radio‐therapies that also rely on elevating cellular production of free radicals for cell killing (Figure 5).

#### 5.1.1 Existing Prooxidant Strategies for Proliferative Disease

Several prooxidants have been used for treating cancers. For example, a prooxidant combination of resveratrol and copper reduced chemotherapy‐associated nonhematological toxicities in advanced gastric cancer in a prospective open label phase II study [212]. Prooxidant drug‐loaded Au/ZnO hybrid nanoparticles have been established as a potential anticancer remedy [213]. The thioreductase inhibitor auranofin was shown capable of selectively targeting cancer stem cells in the treatment of advanced stage cancers by promoting excessive mtROS [214]. The anticancer activity of 7‐O‐pentyl quercetin, a plant‐derived flavonoid, was identified and associated with its prooxidant roles [215]. Direct delivery of free radicals taking advantages of nanotechnologies have also been exploited in treating proliferative conditions, with ROS‐responsive polymeric nanomicelles being fabricated and demonstrated with an outstanding efficacy. For example, DOX‐loaded pH/ROS‐responsive polymeric micelle showed remarkable tumor‐inhibitory capability in vivo by enhancing the ROS level within lung cancer cells [216]. Targeting endogenous antioxidant pathways has also been shown promising in sensitizing cancer cells to conventional therapies. Brusatol, an NRF2 inhibitor, induced the apoptosis of non‐small cell lung cancer cells [217], hindered the progression of bladder cancer cells [218], attenuated the proliferation and invasion of differentiated thyroid cancer cells [219], improved the vulnerability of pancreatic cancer to chemotherapy [220], and sensitized endometrial hyperplasia and cancer to progestin [221].

Prooxidant strategies have also demonstrated therapeutic potential in the treatment of fibrotic diseases (Table 1). The prooxidant sorafenib attenuated liver fibrosis by triggering the ferroptosis of hepatic stellate cells [222], and protected renal fibrosis induced by unilateral ureteral obstruction [223]. Given these preclinical evidence, sorafenib‐loaded silica‐containing redox nanoparticles have been established and proposed as an oral therapy for treating liver fibrosis [224].

#### 5.1.2 CAP for Proliferative Disease

**FIGURE 6:**
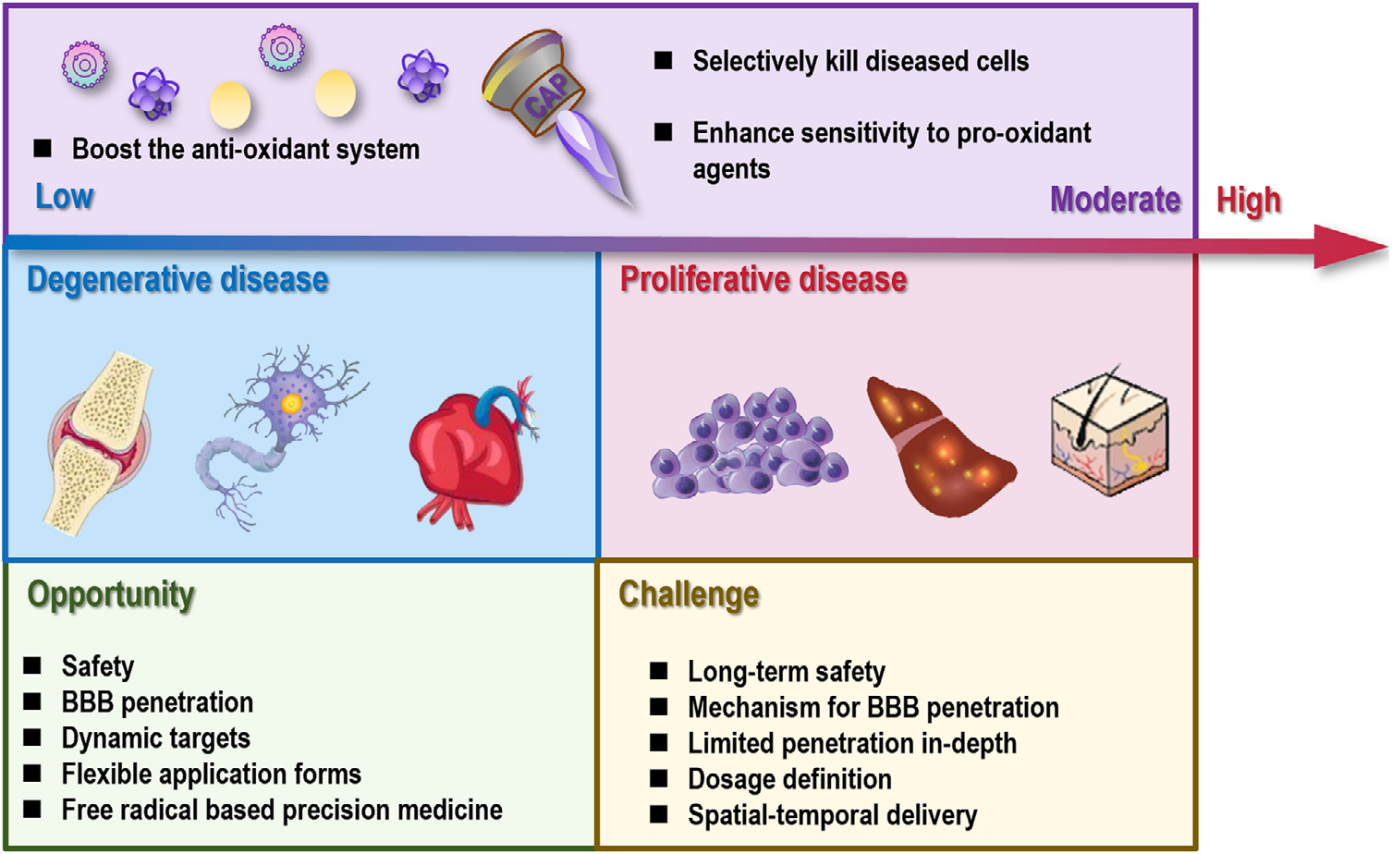
Therapeutic opportunities and challenges of cold atmospheric plasma through redox regulation. CAP can be used to treat both proliferative and degenerative diseases as the concentration of free radicals it contains fall into the low to moderate range (as compared with other pro‐oxidative approaches such as chemotherapy) that can be tuned for desirable outcome. When tuned at low dosages, CAP can boost cells’ antioxidant machinery to restore their cellular redox homeostasis and has thus emerged as an innovative therapeutic tool for treating degenerative diseases such as neurodegenerations such as Alzheimer's disease and Parkinson's disease, joint disorders such as osteoarthritis, and metabolic syndromes like cardiovascular diseases and diabetes. When tuned at moderate dosages, CAP can selectively kill rapidly growing cells and enhance the sensitivity of these cells to prooxidant agents, as these cells have inherently higher basal ROS levels than their healthy peers as a result of elevated metabolic activity and vulnerable antioxidant machinery. Besides these aforementioned unique benefits of CAP in disease management, it also offers opportunities to resolve several critical issues in current medicine. These include the safety of CAP in treating cells given its mildness, the ability of penetrating through the blood–brain barrier (BBB), the dynamic targeting profiles rendering cells less likely developing CAP resistance, the flexible application forms such as CAP‐activated liquids and hydrogel, and free radical based precision medicine through calibrated dosage control and/or synergies with other therapeutics. Yet, challenges pertain, since long‐term safety has not been monitored, the mechanism for BBB penetration remains elusive, direct CAP ejection has limited penetration in‐depth, no consensus on CAP dosage definition, and lack of techniques for spatial–temporal CAP delivery.

One unique benefit of CAP in treating proliferative diseases lies in its selective cytotoxicity against highly proliferating cells [19, 22] (Figure 6). That is, CAP‐derived ROS trigger the death of fast‐growing cells while sparing normal cells. This is because that CAP‐generated RONS exceed the tolerance threshold of diseased cells due to their inherently high basal ROS levels as a result of elevated metabolic activity and collapsed antioxidant machinery. Consequently, the oxidative stress disrupts the mitochondrial membrane potential, leading to various forms of programmed cell death events.

Recent advances have suggested that CAP may initiate different death programs of cancer cells via activating different phosphorylation sites of EGFR. For instance, CAP induced the apoptosis of triple negative breast cancer (TNBC) cells via activating EGFR(Y992/1173) [225], and triggered TNBC ferroptosis via stimulating EGFR(Y1068) phosphorylation [226]. The anticancer efficacy of CAP has also been documented in attenuating the stemness of cancer cells [227], blocking the EMT process and halting metastasis [228]. CAP has also been implicated in modulating the immune system of various types of cancers from diverse aspects such as enhancing the immunogenic cell death of cancer cells [229], polarizing macrophages to the M1 state in the EMT [230, 231], stimulating DCs [232, 233], and enhancing the adaptive immune response [234]. CAP has also demonstrated enhanced overall therapeutic efficacy when combined with existing treatment modalities such as immunotherapy and chemotherapy [235]. For instance, CAP has been combined with checkpoint inhibitors (anti‐PD1) in treating melanoma [236] and proposed as an adjunct to immunotherapy for treating glioblastoma multiforme [237]. In addition, the synergistic use of CAP with temozolomide resulted in enhanced anticancer efficacy in treating glioblastoma [238]; and CAP‐conditioned Ringer's lactate enhanced the cytotoxic activity of cisplatin and gemcitabine in treating pancreatic cancer cells [239]. The first United States Food and Drug Administration‐approved phase I clinical trial examining the efficacy and safety of using CAP for cancer ablation was conducted from March 2020 to April 2021 in the USA (NCT04267575). In this trial, 20 patients carrying various types of metastatic or recurrent solid tumors were recruited and subjected to surgical resections combined with intraoperative CAP treatment. The primary endpoint was safety, and secondary endpoints were patient survival and local recurrence, among others. As the results, local nonrecurrence rate at 28 months was 75%, and no adverse event was reported.

CAP has also been shown promising in treating proliferative syndromes in the field of dermatology. Psoriasis, characterized by the presence of itchy red plaques on the skin, is one of these disorders known sensitive to CAP treatment. Among all possible mechanisms potentiating the efficacy of CAP in treating these disease syndromes, the ability of CAP in inhibiting the hyper‐proliferation of keratinocytes and attenuating abnormal inflammation for restored cytokine homeostasis play the central roles [22, 240]. In support of this, CAP significantly reduced inflammatory cytokines such as IL‐17A, IL‐23A, IL‐24, IL‐1β, and TNFα in psoriasis cells and modulated critical factors in the MAPK pathway in vitro, and led to substantial improvements in factors such as skin thickness, erythema and scaling in vivo [241]. Several clinical success have supported the application of CAP for treating proliferative skin syndromes. In a case report evaluating the effectiveness of CAP in treating a psoriatic plaque on the hand of a 20‐year‐old woman, complete disappearance of the cutaneous lesion was observed after 14 days post‐CAP treatment [242]. In another study, a 26‐year‐old woman with a 3‐month history of inverse psoriasis received CAP treatment for five times at a dosage of 5 min per lesion and a frequency of two to three times per week, and the lesions were almost completely removed with no sign of recurrence during the 6‐week follow up. Also in this study, a 38‐year‐old female with a 4‐year history of scalp psoriasis was treated with CAP for eight times at the same treatment duration per time and therapeutic frequency, and the symptoms were substantially relieved with no adverse reactions nor recurrence according to the 1‐month follow‐up record [243]. Furthermore, randomized trials have substantiated CAP's efficacy, safety, and versatility in treating diverse dermatological disorders including, for example, keloid (NCT04205942) [244], Malassezia folliculitis (NCT04886323) [245], and herpes zoster (ChiCTR2300069993) [246].

### 5.2 Antioxidant Strategies for Degenerative Disease

Degenerative diseases, including neurodegenerative disorders such as Alzheimer's disease and Parkinson's disease, joint degenerative diseases such as rheumatoid arthritis (RA), and metabolic syndromes such as diabetes are among the most significant health challenges globally. These conditions are characterized by the progressive breakdown of tissues and organs, often exacerbated by oxidative stress. There has been a surge of interest in antioxidant strategies as a potential means to mitigate the progression and impact of these diseases (Table 1).

Oxidative stress arises from an imbalance between the production of free radicals and the body's ability to neutralize them with antioxidants. Free radicals can damage cellular components, including lipids, proteins, and DNA, which can lead to the accumulation of misfolded proteins, mitochondrial dysfunction, and inflammation in the context of degenerative diseases. Advances in redox biology have refined our understandings of antioxidant interventions, moving beyond traditional scavengers to targeted therapies that enhance endogenous antioxidative defenses or modulate redox‐sensitive pathways.

#### 5.2.1 Existing Antioxidant Strategies for Degenerative Disease

Dietary intake of nutritional antioxidants has been a focus of recent studies. Foods enriched with vitamins C and E, carotenoids, and polyphenols have shown promise in reducing the oxidative stress. For instance, the benefits of a Mediterranean diet (which is abundant in antioxidant enriched foods such as fruits, vegetables, nuts, and olive oil) in reducing the risk of developing Parkinson's disease [247], RA [248], NAFLD [249], and cardiometabolic diseases [250] have been highlighted. Mitoquinone, a mitochondria‐targeted antioxidant, protected corneal endothelial cells from oxidative damages and improved the mitochondrial bioenergetics [251, 252]. Coenzyme Q10 (CoQ10) was proposed beneficial for individuals suffering from chronic ischemic myocardium as evidenced from the enhanced expression of mitochondrial antioxidant proteins in a swine model of hibernating myocardium [253].

Lifestyles such as physical exercises play a crucial role in modulating the oxidative stress. Regular physical activity has been shown to increase the expression of antioxidant enzymes and thus reduce inflammation. It has been found that moderate‐intensity exercise could improve the cognitive function in individuals with mild cognitive impairment via reducing the oxidative stress according to a randomized controlled trial [254]. On the other hand, habits such as smoking [255, 256] and alcohol abuse [257] or prenatal exposure [258] have been identified as significant contributors to the oxidative stress and the development of degenerative diseases such as cognition impairment.

Pharmacological antioxidant interventions such as NRF2 activators and SIRT1 activators have shown a great potential via enhancing the expression of endogenous antioxidant enzymes. Sulforaphane, a compound derived from cruciferous vegetables and considered as a NRF2 activator, has been investigated for its ability in reducing oxidative damages in the neurodegenerative models of Alzheimer's disease [259, 260, 261] and Parkinson's disease [262, 263]. As another example of NRF2 activator, bardoxolone methyl, a synthetic triterpenoid, ameliorated myocardial ischemia [264], relieved hyperglycemia‐induced mitochondrial dysfunction in experimental diabetic neuropathy [265], and ameliorated high glucose‐induced oxidative injury in human umbilical vein endothelial cells [266]. The SIRT1 activator resveratrol improved mitochondrial biogenesis by activating SIRT1/PGC1α signaling in severe acute pancreatitis [2], eliminated autophagosomes in cardiomyocytes [267], and slowed down the pathogenesis of neurodegenerative diseases [268] as well as aging [269]. Another SIRT1 activator, nicotinamide riboside, improved the fertility of mice by stimulating the SIRT1/FOXO3 pathway [270], and delayed cardiac complications by promoting mitochondrial fusion in diabetic hearts via the SIRT1/PGC1α (peroxisome proliferator‐activated receptor gamma coactivator 1 alpha)/PPARα (peroxisome proliferator‐activated receptor alpha) axis [271].

#### 5.2.2 CAP for Degenerative Disease

CAP has emerged as a groundbreaking therapeutic tool for treating a wide range of degenerative diseases including, for example, Alzheimer's disease, Parkinson's disease, osteoarthritis, and metabolic syndromes such as cardiovascular diseases and diabetes. This therapeutic potential is rooted in CAP's ability in boosting the antioxidant machinery of cells and thereby restoring cellular redox homeostasis [240] (Figure 6).

RA is a degenerative diseases featured by chronic inflammation and progressive joint destruction. Fibroblast‐like synoviocytes are one of the most important players in the pathology of RA that secrete inflammatory cytokines and produce a series of degrading enzymes to cause severe bone and cartilage damages. CAP was shown capable of inducing the apoptosis of fibroblast‐like synoviocytes, leading to reduced production of destructive factors such as the receptor activator of NFκB ligand and MMP‐3, as well as decreased generation of inflammatory factors such as NFκB and IL‐6 in vitro [272, 273]. Further investigations reported significantly relieved RA symptoms including synovial hyperplasia, inflammatory infiltration, and angiogenesis after CAP treatment on appropriate dosage calibration in vivo, and the mechanisms‐of‐action was attributed to the ability of CAP in stimulating the self‐antioxidant capability of the surrounding healthy tissue [274]. A more recent study reported a similar efficacy of CAP in treating RA both in vitro and in vivo as well as the safety of using CAP for RA management since no adverse effect such as synovial hyperplasia, angiogenesis, and inflammatory infiltration was observed [275].

Diabetes mellitus is a degenerative disease associated with disrupted metabolic plasticity [276]. A study investigated the effect of CAP on diabetes both in vitro and in vivo, and the results showed reduced levels of blood glucose, glycated GPX, and AGEs, leading to 30% increased GPX enzyme activity and 20% increased antioxidant activity, as well as substantially reduced expression of oxidative stress biomarkers and decreased concentrations of inflammation factors [277]. Parallel evidence from the clinics offers additional evidence to support CAP's utility in tissue regeneration and treating diabetic complications. In a prospective, randomized, patient‐blinded trial (NCT04205942; *n* = 62 wounds from 43 patients), argon‐CAP (eight applications) adjunctive to standard care (SC) significantly accelerated diabetic foot ulcer healing versus SC + placebo, as evidenced by the greater “average wound area reduction” (*p* = 0.03) and reduced “time to >10% area reduction” (*p* = 9E−3), with no treatment‐related adverse event being reported [278]. Similarly, an randomized clinical trial (IRCT20080904001199N2; *n* = 44) using helium‐CAP (10 kV, 6 kHz) three times weekly for 3 weeks alongside SC achieved 50% wound closure in 77.3% of patients versus 36.4% in SC‐only controls (*p* = 6E−3) [279]. More recent clinical data (DRKS00019943) further confirmed CAP's adjunctive value, demonstrating exclusive “complete wound closure” in CAP + SC cohorts alongside significantly reduced “antibiotic usage” (4 vs. 23%) and “wound pain” versus SC alone [280].

Neurodegenerative diseases such as Alzheimer's disease has been positively linked with type 2 diabetes given their shared features such as brain atrophy, reduced cerebral glucose metabolism, and CNS insulin resistance. It has been unveiled that glucose dyshomeostasis, insulin resistance and impaired insulin signaling are promotive to the pathology of Alzheimer's disease as reflected by enhanced accumulation of Aβ and hyperphosphorylated tau [10]. Thus, evidences supporting the efficacy of CAP in treating diabetes are implicative of its roles in treating neurodegenerative syndromes that deserve intensive preclinical investigations and clinical efforts. With continued advancements in research and clinical applications, CAP‐based therapies may soon transit from the laboratory to the clinic, offering a new hope for individuals affected by these degenerative conditions.

In summary, CAP has emerged as a dynamically calibrated redox platform. That is, by delivering targeted pro‐oxidative bursts for proliferative eradication or restorative signaling for degenerative repair, CAP offers therapeutic benefits surpassing static conventional agents that have positioned it as a promising next‐generation precision tool across therapeutic dichotomies.

## 6 Emerging Frontiers

This section identifies several emerging frontiers in redox medicine that converge on precision technologies. Specifically, artificial intelligence (AI)‐driven biomarker/drug discovery deciphers pathological signatures, nanotechnology enables spatiotemporal control of therapeutic delivery, and CAP engineering tailors radical flux. These may collectively overcome traditional limitations in targeting dynamic redox microenvironments across diversified pathologies.

### 6.1 AI‐Driven Redox Biomarker and Drug Discovery

One emerging frontier in redox precision medicine is leveraging AI and machine learning for comprehensively tackling the complexities of redox biology, which is crucial for understanding and treating a vast array of diseases. This is achieved by simultaneously accelerating the identification of novel therapeutic compounds targeting specific redox nodes such as the NRF2 pathway, the GSH system, the Trx machinery, or NADPH oxidases, and enabling the discovery, validation, and clinical implementation of redox‐sensitive biomarkers indicative of disease onset and progression, disease state and therapeutic response, or sensitivity to redox therapeutics. This integrative approach harnesses the power of AI algorithms, spanning deep neural networks, graph neural networks for molecular property prediction, natural language processing for mining scientific literature and electronic health records, reinforcement learning for molecular design, and sophisticated bioinformatics pipelines, to analyze massive, heterogeneous datasets encompassing chemical libraries, high‐throughput screening results, phenotypic readouts, imaging data, and crucially, multiomics profiles including the burgeoning field of single‐cell redoxomics. This will provide unprecedented resolution by quantifying the dynamic redox state, RONS fluxes, antioxidant capacities, and the expression/activity of redox‐sensitive proteins and pathways within individual cells, revealing critical heterogeneity masked in bulk tissue analyses and pinpointing rare pathogenic cell populations or specific cellular compartments driving disease initiation and progression.

Single‐cell redoxomics techniques such as advanced flow cytometry with redox‐sensitive dyes, mass cytometry, highly multiplexed immunofluorescence, spatially resolved transcriptomics/proteomics, and emerging single‐cell metabolomics platforms, generate high‐dimensional data that AI excels at deciphering for the identification of subtle yet significant redox signatures unique to specific cell types or states within complex tissues. This will lead to the uncover of novel, context‐specific redox biomarkers with enhanced diagnostic and prognostic power, and the reveal of new druggable targets within defined cellular subsets to enable the prediction of cell‐type‐specific drug effects and potential mechanisms leading to therapeutic resistance. Consequently, AI models trained on integrated single‐cell redoxomic data and chemical/biological databases can rationally design, virtually screen, and optimize next‐generation redox‐modulating drugs with improved selectivity for pathological redox alterations in specific cells, enhanced pharmacokinetic properties, and reduced off‐target effects. In addition, this will concurrently identify companion redox biomarkers for patient stratification and treatment monitoring. This powerful synergy between AI, single‐cell resolution redox analysis, and biomarker discovery is overcoming the historical challenges of redox drug development such as target promiscuity, cellular context‐dependency, and the delicate balance between therapeutic oxidative stress induction and protection. This will ultimately usher in a new era of precision redox medicine where therapies and diagnostics are tailored to the intricate and heterogeneous redox landscapes that operate at the fundamental level of individual cells within living systems to fundamentally advance our ability in modulating the “redoxome” for the benefits of precision medicine.

### 6.2 Nanotechnology‐Based Spatial–Temporal Control of Redox‐Agent Delivery

Nanotechnology‐based spatial–temporal control of redox‐agent delivery represents a revolutionary frontier in redox precision medicine. This leverages meticulously engineered nanocarriers including liposomes, polymeric nanoparticles, dendrimers, micelles, inorganic nanoparticles, and hybrid nanostructure, to achieve unprecedented precision in governing where, when, and how much therapeutic redox‐modulating agent is released within the complex biological milieu. This can overcome the critical limitations of systemic administration that lead to off‐target effects, inadequate bioavailability, rapid clearance, dose‐limiting toxicity, and failure to reach therapeutically relevant concentrations at the precise diseased site. Spatial control is engineered through passive targeting strategies exploiting the enhanced permeability and retention effect prevalent in pathological tissues or inflamed sites, and active targeting mechanisms where nanocarriers are decorated with specific ligands to recognize overexpressed receptors uniquely present on the surface of target cells or within the diseased microenvironment. Temporal control is orchestrated through the design of stimuli‐responsive nanocarriers that remain inert during circulation but undergo triggered payload release once encountering specific endogenous or exogenous stimuli intrinsic to the pathological niche. Endogenous stimuli‐responsive systems exploit the unique biochemical signatures of diseased sites such as low pH, elevated levels of specific enzymes, and abnormal redox potential gradients, causing nanoparticle destabilization or conformational changes for the precise liberation of encapsulated redox cargo within the target tissue. Exogenous stimuli‐responsive systems offer even finer external spatiotemporal command, utilizing externally applied triggers such as near‐infrared light, magnetic field, ultrasound, or mild alternating radiofrequency to induce highly localized, on‐demand payload release at predetermined time points and specific anatomical loci with minimal background leakage. Such a sophisticated dual control paradigm enables not only the protection of sensitive redox agents from premature degradation but also the sequential or simultaneous delivery of multiple agents with precise kinetic profiles, allowing for the mimicking of natural redox signaling dynamics or the creation of sophisticated therapeutic windows with profound clinical implications across diverse pathologies. In proliferative diseases such as cancer, it allows tumor‐specific delivery of prooxidant drugs to induce lethal oxidative stress in malignant cells while sparing healthy tissue, or targeted delivery of antioxidant enzymes to mitigate chemotherapy‐induced off‐target toxicity. In degenerative syndromes such as Alzheimer's disease, it facilitates blood–brain barrier (BBB) penetration and neuron‐specific delivery of neuroprotective antioxidants to combat against the oxidative damage.

Furthermore, the integration of imaging moieties (e.g., fluorescent dyes, magnetic resonance imaging [MRI] contrast agents) within these nanoplatforms allows for real‐time, noninvasive tracking of nanoparticle biodistribution and payload release kinetics (theranostics), enabling treatment monitoring and dose optimization. Despite challenges in scalability, long‐term biocompatibility assessment, complex manufacturing, and navigating regulatory pathways, the unparalleled ability of nanotechnology to provide spatiotemporally resolved redox modulation heralds a transformative era in treating diseases rooted in redox dysregulation with unprecedented efficacy and safety.

### 6.3 CAP Precision Engineering

Given the dual roles that free radicals play in human health and disease, improper dosing of CAP may pose safety concerns especially for long‐term use (Figure 6). Several pre‐ and clinical studies have reported the safety of CAP for different medical purposes. For instance, 5 min CAP treatment in the upper respiratory tract did not cause any cell damage in vitro, yet long‐term effects of CAP on the organism needs to be carefully examined in vivo [281]. A prospective, randomized control trial documented the efficacy and safety of using CAP in the treatment of keloids, with only one small scab reported in one out of 18 patients recruited [244]. In another clinical trial examining the efficacy and safety of using CAP for treating striae distensae, 52.3% patients rated satisfaction as a great improvement, 39.1% as moderate improvement, and 4.3% as extreme improvement, with minimal side effects reported [282]. However, given the dual role of CAP played in health management and its dose‐dependent nature, long‐term monitoring on individuals receiving CAP treatment is essential, where biomolecule‐based indicators for different medication outcomes and safety concerns are needed to be established. Accordingly, large‐scale clinical trials designed to monitor long‐term outcomes are necessary to fully establish the safety profile of CAP in the clinical practice.

CAP has demonstrated remarkable potential in transiently and reversibly disrupting the BBB, a major obstacle in treating CNS disorders such as neurodegenerative diseases and brain cancers [283] (Figure 6). This transient BBB opening enhances the delivery of therapeutics that are otherwise excluded by the barrier's restrictive nature. On one hand, CAP‐generated reactive species may modulate BBB permeability by inducing the internalization and disassembly of endothelial tight junction proteins such as claudin and occludin through reversible oxidation. In consistent with this, low dosage of CAP reduced claudin‐2 expression and protein stability in human lung adenocarcinoma cells [284]. On the other hand, CAP may degrade ECM and the basal lamina by activating MMPs. In support of this, CAP modified the ECM to influence chondrogenesis and endochondral ossification [285]. Last, CAP may enhance the blood flow and convective drug transport into the brain by triggering vasodilation. Specifically, NO• within CAP may help relax tensions in the blood vessels by activating the sGC–cGMP axis [286]. Intensive experimental validations are needed to consolidate these hypothetical mechanisms before efforts are engaged to exploit CAP for enhanced drug delivery through the BBB. Also, real‐time monitoring including the development of optical coherence tomography or MRI‐based systems to visualize BBB opening dynamics during CAP treatment may further refine the clinical utility of CAP, bridging the gap between preclinical promise and patient care.

Unlike targeted therapies where the therapeutic targets are fixed, CAP addresses imbalanced redox system that is a multifaceted network regulated by concurrent pathways and cell‐specific molecular panels. Thus, unless the cellular system is rewired back to the homeostatic state, CAP remains effective in dynamically fixing errors inducing redox chaos. In other words, CAP can be viewed as a dynamic systemic agent targeting dysregulated redox equilibrium, inherently minimizing the development of therapeutic resistance in diseased cells (Figure 6).

The limited penetration in‐depth of CAP through direct ejection, that is, approximately 2 mm, has largely restricted its application to superficial lesions. Endoscopic probes equipped with CAP delivery systems have been explored to target deeper tissues directly, expanding the range of treatable lesions [287]. Importantly, CAP is highly flexible regarding its clinical use, allowing for diverse delivery forms to suit various medical needs (Figure 6). CAP can be administered as a liquid, hydrogel, or aerosol, each offering unique advantages. For instance, CAP‐activated liquids such as Ringer's lactate solution [239] and phosphate buffered saline [288] as well as hydrogel formulations [289] can be directly applied to the disease lesions or integrated into intravenous therapies. CAP‐activated aerosol [290, 291], on the other hand, can reach remote or hard‐to‐access areas, such as the respiratory tract or internal cavities, making it suitable for applications like lung infections or minimally invasive surgeries. Such a versatility of CAP regarding its delivery forms can substantially enhance the potential of CAP in addressing a wide range of clinical challenges, from surface disinfection to deep tissue therapy. Yet, short‐lived species such as OH· and O_2_^•^ can hardly be preserved within liquids, hydrogels or aerosols given their transient lifespans. Thus, resolving such a critical challenge taking advantages of, for example, nanotechnologies, may truly broaden the therapeutic range of CAP in modern medicine.

CAP represents a paradigm shift in health and disease management by leveraging free radicals for selective ablation of abnormal cells at calibrated dosages and creating synergies with conventional therapies. Recent advances have highlighted its medical potential in preclinical and early clinical settings, yet challenges in long‐term safety and penetration in‐depth require appropriate solutions. With innovations in nanotechnologies [292, 293], AI and high‐throughput sequencing, CAP‐based therapies are poised to transform precision medicine, offering a noninvasive, cost‐effective alternative to traditional modalities (Figure 6). For example, combining CAP with gold nanoparticles functionalized with phosphorylated focal adhesion kinase antibody eradicated approximately half oral squamous cell carcinoma cells [294], suggesting enhanced free radical delivery and anticancer effects. ROS‐responsive nanoparticles may help enhance the spatial precision of CAP for minimized off‐target effects by improving T1‐MRI imaging contrast for tumor‐specific detection, allowing for precise drug release and improved treatment efficacy. AI‐driven CAP devices capable of adjusting free radical profiles based on tumor redox status such as the GSH/GSSG ratio are promising in optimizing the treatment efficacy. High‐throughput sequencing including that at the resolution of the single‐cell level may unveil the impact of CAP in reshaping the immune response landscape, tumor microenvironment and the tumor–immune crosstalk, highlighting the potential for developing tailored combination therapies that optimize treatment outcomes by aligning therapeutic strategies with individual tumor profiles.

In summary, the synergy of AI‐predictive analytics, nanotechnology‐mediated spatial precision, and CAP's tunable bioactivity will establish a next‐generation framework for redox therapeutics. This triad may synergistically shift paradigms from empirical dosing to mechanism‐guided dynamic intervention, which is poised to redefine precision medicine.

## 7 Conclusions

Free radicals are indispensable in relaying cellular signals and sensing redox signals for the maintenance of mitochondrial and immune homeostasis, deficient supply of which imposes cells with the reductive stress and is associated with a plethora of degenerative syndromes. Yet, free radicals are dangerous that can cause damages to cellular materials, overproduction of which sets cells to the oxidative stress that may potentiate proliferative diseases especially cancers. The dual roles of free radicals in nature highlight the necessity of maintaining redox homeostasis for the sake of keeping health and managing disease. Free radicals are primarily produced during mitochondria respiration and in response to hyperglycemia that characterize the centrality of O_2_ and metal ions during ROS production. Cellular redox balance is subjected to a multilayer regulation involving antioxidant systems and signaling framework, as well as damage repair machinery and dynamic cellular compartment interplay.

It is worth noting that current antioxidant therapies have not translated well in the clinics. For instance, clinical trials investigating antioxidants for Alzheimer's disease prevention or treatment have yielded predominantly negative outcomes, underscoring the critical importance of redox intervention dosing [11]. A 16‐week double‐blind trial targeting cellular compartments with vitamins E/C + α‐lipoic acid (E/C/ALA) or CoQ in mild‐to‐moderate Alzheimer's disease patients demonstrated accelerated cognitive decline in the E/C/ALA group, revealing significant safety concerns despite reduced oxidative stress biomarkers [12]. Similarly, an open‐label trial of a multi‐ingredient formulation (folic acid, B12, vitamin E, S‐adenosyl methionine, NAC, acetyl‐l‐carnitine) showed cognitive benefits exclusively in nondemented younger adults, with no efficacy in older cohorts (>74 years), highlighting age‐dependent therapeutic windows [13]. Ophthalmic studies further corroborated this risk profile, with vitamin E/C/β‐carotene supplements being associated with increased incidence of developing both early and late age‐related macular degeneration [14, 15]. Beyond degenerative diseases, high‐dose vitamin A/β‐carotene supplementation in the clinics increased lung cancer mortality risk (RR = 1.16, 95% CI = 1.06–1.26) after 4 years [16, 17], whereas the use of peri‐chemotherapy vitamin A elevated breast cancer recurrence (HR = 4.23, 95% CI = 1.32–13.57) and death (HR = 3.53, 95% CI = 1.05–11.93) [18]. Collectively, these findings have validated that excessive antioxidants might induce the reductive stress, paradoxically acting as prooxidants to cause cellular damages [295, 296], whereas subtherapeutic doses might fail to modulate advanced disease pathologies.

Though degenerative and proliferative pathological conditions are both subjected to redox imbalance where either prooxidant or antioxidant approaches may apply under certain conditions, antioxidant therapies dominate degenerative disease management and prooxidant approaches are gaining traction in treating proliferative syndromes especially cancers. These have necessitated the possibility of curing various health concerns with one therapeutic modality that could spatiotemporally control the concentrations of free radicals within cells.

Innovations like CAP may represent this therapeutic hope and exemplify this potential medical paradigm shift toward fine‐tuned radical modulation. By generating controlled levels of reactive species, CAP has shown a great promise in boosting the antioxidant machinery of cells for treating degenerative diseases, with demonstrated efficacy in modulating redox signaling, enhancing tissue repair, and mitigating inflammation. On the other hand, CAP can selectively target rapidly growing cells while sparing healthy tissues, offering an unique approach in treating proliferative conditions such as cancers. Besides, CAP can aid in the BBB penetration process, enabling efficient drug delivery into the brain and providing extra therapeutic advantages. These unique traits enable precise tuning of the radical flux of CAP to disease‐stage‐specific pathophysiology across the health‐to‐disease continuum. Further investigations are needed to overcome the limited penetration depth of CAP direct ejection, to address its long‐term safety and achieve precise dosage control before it can be truly translated into clinical applications. This may be achieved through integrating knowledge from other research domains such as nanotechnologies, AI, and omics information retrieval.

Advances in redox biology may offer novel strategies to harness free radicals for therapeutic benefits beyond CAP. Future research should focus on personalized redox therapies to balance the protective and detrimental effects of free radicals in human health. Specifically, the development of biomarker‐driven dosing protocols will be of critical values for optimizing therapeutic outcomes while minimizing the possible adverse effects. Alongside, rigorous assessment of disease‐stage redox signatures and calibrated control over radical kinetics and spatiotemporal delivery are also needed. Additionally, understanding and harmonizing the interplay between endogenous and exogenous sources of free radicals may be essential for tailoring the intervention strategies for a particular individual. This includes exploring how environmental factors, lifestyle choices, and genetic predispositions influence redox homeostasis and disease progression. Importantly, By integrating these approaches, future research may unlock the full potential of redox modulation for human health and disease management.

## Author Contributions

X. Dai conceived the idea, prepared the manuscript and figures, as well as provided the financial support. Z.Z. Huang and R. Lyu contributed in figure and table preparation. All authors have read and approved the final manuscript.

## Conflicts of Interest

The authors declare no conflicts of interest.

## Ethics Statement

The authors have nothing to report.

## Data Availability

The authors have nothing to report.
